# Lexical bundles in psychology lectures and textbooks: a contrastive corpus-based study with implications for academic writing

**DOI:** 10.3389/fpsyg.2025.1545355

**Published:** 2025-04-14

**Authors:** Abdullah Alasmary

**Affiliations:** Department of English Language and Translation, College of Language Sciences, King Saud University, Riyadh, Saudi Arabia

**Keywords:** psychology register, lexical bundles, corpus analysis, register variation, writing instruction

## Abstract

Research on lexical bundles (LBs) has explored various academic domains; however, the field of psychology has received comparatively less attention. This study aims to address this gap by contrastively investigating the use of LBs in two sub-corpora: videotaped lectures and textbook chapters. Four-word bundles that meet a predetermined set of selection criteria are elicited and subsequently analyzed both structurally and functionally. The results indicate significant variation in the number of bundle types and tokens between the two registers, with the spoken register exhibiting a much broader variety of LBs than the written register. Structural analysis reveals that clausal constructions predominantly characterize LBs in the spoken psychology register, whereas phrasal patterns are more common in the written register. Additionally, the functional analysis highlights that stance bundles constitute the most prevalent category in the academic lecture corpus, while referential bundles emerge as the largest functional category in the academic texts. This variation reinforces the widely accepted notion that LBs are sensitive to register differences. Pedagogically, the study provides English for Specific Purposes instructors with data-based lists of LBs that can be integrated into classroom activities or tailored to develop instructional materials on academic writing and speech. Given that LBs are classified into distinct structural and functional groups, moreover, instructors can draw on the two lists for more register-focused, awareness-raising activities that help psychology students approximate an expert-like writing style.

## 1 Introduction

There is a broad consensus among scholars that lexical bundles (LBs) play a critical role in knowledge creation and dissemination, serving as markers of linguistic proficiency. Defined as “extended collocations, sequences of three or more words that statistically co-occur in a register” (Cortes, 2004, p. 400), LBs are recognized as “an important component of fluent linguistic production” (Hyland, 2012) and function as the “building blocks of discourse” (Biber et al., 2004, p. 371). These patterns have been studied under various terminologies, including *phraseology* (Le and Harrington, 2015), *word combinations* (Ädel and Erman, 2012), *formulas* (Simpson-Vlach and Ellis, 2010), *multiword constructions* (Liu, 2012; Wood and Appel, 2014), and *multiword units* (Hoang and Crosthwaite, 2024). While these terms are sometimes used interchangeably (e.g., Le and Harrington, 2015; Vincent, 2013), they exhibit differences in terms of conceptualization and identification criteria. The notions of phraseology and formulas emphasize the conventionalized meaning and the pedagogical usefulness of patterns emerging from corpus analysis (e.g., Simpson-Vlach and Ellis, 2010), whereas multiword constructions are used in cognitive linguistics to describe structured, non-arbitrary linguistic units that co-occur together, fulfilling a specific discourse function Liu (2012). Lexical bundles, as conceptualized by Biber et al. (1999), are strictly identified according to frequency rather than semantic transparency, grammatical completeness, or pedagogical utility.

Research has underscored several defining characteristics of LBs. First, these recurring patterns facilitate faster processing and comprehension compared to arbitrarily assembled sentences (Tremblay et al., 2011). Second, LBs exhibit variability in their grammatical forms and discourse functions (Biber et al., 2004; Hyland, 2008a, 2008b), necessitating detailed analyses to understand how scientific communities produce and disseminate specialized knowledge. Third, the use of LBs is influenced by factors such as discipline (Cortes, 2004), the professional or academic backgrounds of writers (Esfandiari and Barbary, 2017), and genre or register (Biber and Barbieri, 2007). Finally, LBs are “highly sensitive to differences between text types” (Durrant, 2017, p. 166), with their frequency, structure, and functions aligning closely with the communicative purposes and situational features of specific texts.

Over recent decades, corpus-based studies have systematically investigated the distributional, structural, and functional characteristics of LBs across diverse disciplinary domains and text types. Findings suggest that each discipline favors distinct bundles that reflect the rhetorical preferences and community-approved conventions unique to that field. For instance, *the cost of the* (Wood and Appel, 2014) is commonly found in business discourse, while *if and only if* (Author, 2019) dominates mathematical texts. Such discipline-specific patterns underscore the need to explore underrepresented fields, such as psychology, to better understand how LBs function within their unique linguistic contexts. Despite the significant progress that has been made on the study of LBs in a wide range of different disciplines, psychology remains practically under-researched compared to other academic fields (Farhang-Ju et al., 2024; Le and Harrington, 2015; Qin, 2014; Ren, 2021). Previous studies have thus focused on comparing the use of LBs by native English-speaking psychologists and non-native psychology authors in published research articles (Esfandiari and Barbary, 2017), revealing considerable variation in the structural and functional properties of these patterns across native and non-native academic writing. Durrant (2017) used a corpus of student writings to situate psychology within broader spectrum of disciplines, indicating the interdisciplinary nature of psychology as it draws on science/technology and humanities/social sciences. Although these studies have deepened our understanding of LBs in the research articles and disciplinary writings, there is a need to extend this line of inquiry and address other psychology registers which have been overlooked.

The present study addresses this gap by using corpus-informed approaches in identifying lexical bundles in two psychology sub-corpora: academic lectures and written textbooks. By contrasting spoken and written registers within the field of psychology, this research seeks to shed light on the register-specific language patterns and their pedagogical implications for the teaching and learning of psychology. A primary goal of the study is to provide psychology writers, educational material developers, and English for Specific Purposes (ESP) instructors with a comprehensive inventory of LBs that can inform the writing practices of psychology students. Using authentic data from both registers, the study offers valuable insights into the community-authorized ways of using language in psychology, reveals patterns of expert discourse, and advances evidence-based pedagogical approaches to the linguistic analysis of psychology registers.

## 2 Literature review

### 2.1 LBs across disciplines and genres

Research into LBs is multifaceted and multidimensional, seeking not only to understand the grammatical and functional attributes of such patterns, but also to determine their role in the construction and dissemination of knowledge across disciplines, genres, and registers. A great body of research has thus focused on the use of such patterns in specific domains such as history and biology (Cortes, 2004), telecommunications (Pan et al., 2016), business and engineering (Wood and Appel, 2014), applied linguistics (Le and Harrington, 2015; Qin, 2014; Ren, 2021; Shirazizadeh and Amirfazlian, 2021), law (Breeze, 2013), mathematics (Author, 2019), pharmaceutical studies (Grabowski, 2015; Ren, 2021), and psychology (Esfandiari and Barbary, 2017). These discipline-focused studies seem to suggest that while there are some LBs which transcend disciplinary boundaries, the majority of LBs are discipline-specific. Bundles such as *in the treatment of* (Grabowski, 2015), *the performance of the* (Pan et al., 2016), *the rule of law* (Breeze, 2013), *the design of the* (Wood and Appel, 2014), and *the concept of the* (Le and Harrington, 2015) are examples of field-specific LBs, whereas bundles such as *the nature of the, on the other hand,* and *it is clear that* show a tendency to transcend disciplinary boundaries. In addition to this growing interest in the distribution of bundles in distinct domains, registers, and discourse settings, LBs have also been used as an analytical tool to investigate a wide array of topics. For example, LBs are extracted from various writing samples to draw boundaries separating different domains (Durrant, 2017), uncover the formulaic nature of moves in a disciplinary sub-genre (Cortes, 2013), and to examine instances of definiteness marker misuse by second language learners (Shin et al., 2018). LBs have also been used to explore the role of repeated input for language acquisition (Northbrook and Conklin, 2019), and to identify the degree of fixedness/variability characterizing multiword expressions (Ren, 2021). A considerable number of research studies have taken a pedagogical approach, thus synthesizing and analyzing lexical bundles in different domains with the aim of providing instructors and materials designers with data-informed resources for use in English for Academic/Professional Purposes settings (Author, 2019; Martinez and Schmitt, 2012; Rogers et al., 2021; Simpson-Vlach and Ellis, 2010). Several parameters are applied in the process of obtaining such bundles from language data, often combining corpus analysis with opinion gleaned from experts in producing the final list of the target items.

Another interesting area of research into LBs has addressed the similarities as well as the differences in using such units by native and non-native English writers, resulting in incongruent findings. Ädel and Erman (2012) and Chen and Baker (2010) contrasted the use of LBs in the writings produced by Swedish and Chinese students against similar writing samples produced by native English writers. The result shows that native speakers make use of the widest range of bundle types and tokens in comparison with their L2 peers. However, the analysis of LBs in two parallel corpora comprising argumentative essays by native and non-native students has shown no substantial differences between these two groups (Shin, 2019). Most notably, the two groups appear to use similar proportions of bundle forms and functions.

More recently, research has explored other areas such as the use of LBs in rhetorical moves of the introduction section of journal articles (Farhang-Ju et al., 2024), proximity construction in popular TED talks (Wang and Csomay, 2024), the potential affordances of bundle-based instruction in teaching prepositions to non-native speakers (Kang et al., 2024), the learners’ awareness of genre-specific bundle use among non-native learners (Shin and Won, 2024), and the distribution of bundles on the proficiency scale of high-stakes exam takers (Saadatara et al., 2023). The study by Farhang-Ju et al. (2024) has identified LBs associated with specific communicative moves within the introduction sections of 1,000 annotated journal articles, revealing that some bundles are move-specific, whereas others are more general, occurring across multiple moves. Another important finding is that bundles performing a text-organizing function is the dominant functional group compared with referentials and stance markers. Using LBs to teach prepositions to non-native writers is the purpose of the study by Kang et al. (2024) who demonstrated that LBs can be used as a pedagogical tool to inform the teaching of English prepositions. To gauge the L2 learners’ awareness of genre differences in the use of lexical bundles, Shin and Won (2024) pointed out that variation in the use LBs can be interpreted by genre differences. Learners’ proficiency plays a similarly important role, as demonstrated by Saadatara et al. (2023) who analyzed the forms and functions of LBs in the writing section of the TEOFL exam and found that highly proficient exam takers tend to use more varied forms and functions. Low-proficiency group of exam takers use less varied LBs with restricted forms and functions.

### 2.2 LBs in spoken and written registers

Few studies have examined the use of lexical bundles in oral registers compared with those focusing on written registers. Biber et al. (2004) identified lexical bundles across four university registers: conversation, classroom teaching, textbooks, academic prose. Conversation and classroom teaching represent the oral register, whereas textbooks and academic prose are analyzed as instances of the written register. Results show a substantial degree of inter-register variation in the use of lexical bundles not only in the total number of lexical bundles but also in the functions of such patterns. Spoken registers make use of a greater number of lexical bundles in comparison with the written language of textbooks and academic prose. Functionally, bundles gleaned from the oral corpus are primarily used to express stance or maintain interpersonal interactions, while patterns derived from written registers are functionally more oriented toward disseminating information. In a more extensive study, Biber and Barbieri (2007) included more registers to analyze while maintaining the same purpose of unveiling the structural as well as the functional attributes of four-word lexical bundles. Oral academic registers included classroom teaching, classroom management, office hours, study groups, and service encounter, whereas the written academic register comprised chapters of textbooks, course management, and institutional writing. The analysis of the results maintained that bundles are more prevalent in spoken academic registers than in written academic registers. The number of bundles varies within each register, revealing greater concentration of patterns in classroom management and course management documents. This result is unsurprising, given that the nature of management documents is more formulaic and less compositional. Analyzing lexical bundles in academic lectures was the objective of a study by Nesi and Basturkmen (2006) who found little variation in the use of lexical bundles between American and British academic contexts, but alluded to a distinction that needs to be established between “oral” bundles and “literate” bundles, as the former denotes patterns commonly used in everyday conversation (e.g., do you want to), while the latter refers to elements typical of written academic discourse (e.g., the nature of the). Csomay (2013) investigated the presence as well as the functional use of 84 bundles in a wide range of classroom sessions at various academic institutions. Findings reveal that stance expressions are used more extensively at the beginning of classroom discussion, but referentials and discourse organizers resurfaced as the class discussion continues to progress. Knowledge of such patterns, the study concludes, is expected to aid comprehension and help understand the rhetorical shifts and turns characterizing the spoken genre. In a recent study, Shin and Won (2024) demonstrated that the same group of L2 learners used different sets of lexical bundles once in writing and subsequently through speech although the topic of both is strictly identical. Researchers interpreted these findings by alluding to growing awareness among these L2 learners of appropriate genre-specific conventions.

### 2.3 LBs in psychology registers

Despite the extensive scholarly activity on LBs in various academic disciplines, psychology receives little scrutiny. Yang (2022) built a corpus of journal articles to explore the extent to which LBs are used to establish rhetorical moves within the different parts of the article, namely the introduction, methods, results, and discussion (IMRD). The analysis of results shows that LBs are distributed unevenly across the IMRD sections, with the Discussion section comprising the largest number of bundle types and tokens and the Introduction the fewest. The functional distribution of LBs shows some variations, too. Research-oriented bundles are prevalent in the Methods and Results sections, while text-oriented and participant-oriented bundles dominate the Introduction and Discussion sections. Although Yang (2022) bridges the quantitative analysis of bundles with the qualitative analysis of rhetorical moves, it overlooks other non-IMRD sections, limiting the generalizability of findings within the genre of journal article writings. Lake and Cortes (2020) examined the extent to which LBs reflect disciplinary conventions in Spanish and English academic writing, focusing on literary criticism, history, and psychology. Analysis of patterns emerging from the data reveals that different disciplines exhibit distinct norms of use, with psychology employing more methodological and empirical reporting bundles. In a similar vein, psychology has been the focus of a study by Esfandiari and Barbary (2017) who conducted a corpus-driven contrastive analysis of LBs in research articles written in English by two groups of native and non-native English authors. An important finding is that non-native Persian-speaking psychology authors rely on a limited number of LBs than native peers who employed a broader range of different LBs. Moreover, the study also unveils several instances of first language interference where Persian authors exhibit a higher frequency of unnatural patterns such as *the aim of this study* and *as it can be seen in Figure.* In contrast, native authors produce more concise and direct forms such as *this study aims to* and *as shown in Figure*. Comparative studies on LBs which involve psychology and other disciplines are rare. The study by Cao (2021) compiled a corpus of research articles in psychology and education, examining the impact of research methodology paradigms (quantitative, qualitative, or mixed method) on the use of LBs. Disciplinary differences emerged in both the structure as well as the functions of LBs, with psychology articles comprising more text-oriented bundles to frame arguments, while education has employed more research-oriented bundles to describe methodology and highlight findings. Variation in LBs use is also reported across methodological paradigms. Quantitative research studies favored verb-based LBs which serve a participant-oriented function with the aim of maintaining an objective tone, whereas qualitative papers relied more on preposition-based, text-organizing LBs that may help contextualize arguments. Research papers adopting a mixed method approach exhibited a balanced mixture of structures and functions.

Given that prior research has focused on research papers and student writing, this study is unique as it sheds light on the distributional characteristics of LBs in two unexplored psychology registers: university textbooks and academic lectures. They represent key channels through which disciplinary knowledge is created, disseminated, and interpreted. Through a combination of linguistic analysis and corpus treatment of data, the study aims to seek answers to the following research questions:

1. Which lexical bundles occur most frequently and are most widely distributed in a corpus of introductory university textbooks?
2. Which lexical bundles occur most frequently and are most widely distributed in a corpus of introductory psychology lectures?
3. What are the structural and functional characteristics of LBs in each corpus? Which LBs are register-specific? Which LBs are register-transcending?

## 3 Methods

This study draws on purpose-designed, similarly-sized corpora of authentic language data. In the following section, a detailed description is provided of the processes of creating, refining, and analyzing the study corpora. Next, the procedures for bundle extraction and filtering are outlined. Finally, a brief overview is given on the grammatical and functional analyses of bundles emerging from the corpus analysis.

### 3.1 Corpora

The current study compares the use of LBs in oral and written registers within the domain of psychology. The written corpus comprises chapters from five full-length introductory textbooks focusing on psychology as an academic discipline. These textbooks were obtained from electronic databases accessible through an institution-based subscription (Hsu, 2014). The selection of these textbooks is guided by the following principles:

*Authorship*: The textbooks included in the corpus were authored by researchers or writers affiliated with academic institutions. Books written by non-academic authors without academic affiliations were excluded. The decision to control for the academic background of authors is to ensure consistency with lectures which were all delivered by academic professionals.

*Thematic focus*: Recognizing the multifaceted and multidimensional nature of psychology, the study prioritized introductory textbooks that address a wide range of psychology topics rather than those with a narrow focus on specific themes and sub-fields. Textbooks aimed at general audiences or those centered on specific sub-topics within psychology were excluded from the analysis.

*Publication date*: To ensure relevance and consistency, the textbooks were published within a relatively narrow timeframe (2013–2017). Including textbooks published across widely varying periods of time could raise some concerns due to the rapid evolving nature of psychology.

The spoken corpus is compiled from four courseware platforms: Open Yale Courses (25 lectures), MIT Open Courseware (24 lectures), Stanford University Collection (24 lectures), and UC Berkeley (9 lectures). The videos included in the corpus feature similar titles such as *Introduction to Psychology, Introduction to General psychology,* and *Human Behavior Psychology*. The nature of these recordings is predominantly instructor-centered with few instances of students’ talk. The choice of specific universities is based on their prominence in psychology education, ensuring the inclusion of sample lectures from institutions with well-established psychology programs. A second factor is the presence of these lectures on open-access platforms, allowing the data to be sourced from freely available and widely used materials for psychology instruction. Yet a third factor influencing the selection of lectures from prominent universities is the widely held belief that these institutions maintain rigorous academic and pedagogical standards, serving as exemplars of established conventions in psychology education. All videos are predominantly instructor-led, with minimal instances of student interaction. These recordings were produced in actual classroom settings and were not intended specifically for dissemination on online platforms. To enable corpus analysis, all videotaped lectures were transcribed using an online transcription service.

The two corpora are matched in their thematic focus on the domain of psychology and the number of running words (tokens). However, there are differences which merit some discussion. The written corpus spans approximately 5 years, as is indicated by the publication date. In contrast, there is no specific information regarding the time when the spoken materials were exactly recorded. Another important difference is that each chapter in the written corpus exhibits distinct thematic unity. The thematic unity of each recording is less prominent as shifting from one topic to the other is a distinct characteristic of recorded lectures. Components of each corpus are outlined in Table 1.

**Table 1:** Corpora components.

Corpus	Sub-corpus	# of word types (%)	# of tokens (%)	Type/token ratio
Spoken	Berkeley	4,540 (13.23)	62,601 (9.90)	0.0725
	MIT	10,222 (29.78)	256,492 (36.46)	0.039
	Stanford	10,198 (29.71)	219,162 (31.15)	0.046
	Yale	9,366 (27.29)	166,276 (23.64)	0.056
*Total*			*703,476 (100)*	
Written	Textbook 1	14,924 (22.45)	144,637 (20.56)	0.103
	Textbook 2	13,290 (19.99)	133,947 (19.04)	0.099
	Textbook 3	10,695 (16.09)	121,948 (17.34)	0.087
	Textbook 4	12,443 (18.72)	147,089 (20.91)	0.084
	Textbook 5	7,517 (11.31)	91,927 (13.07)	0.081
	Textbook 6	7,611 (11.45)	63,928 (9.09)	0.119
*Total*			*704,531 (100)*	

### 3.2 LBs selection criteria

Three important criteria are applied in selecting bundles from each corpus: length of the target bundle, frequency of occurrence, and dispersion across the corpus sub-parts. Regarding the length, bundles comprising four words are chosen for analysis. Four-word lexical bundles “hold three-word bundles in their structures” and “present a wider variety of structures and functions to analyze” (Cortes, 2004, p. 401). The frequency of occurrence is the second parameter guiding the process of selecting LBs from both corpora. There is also no consensus on a specific frequency threshold that determines which bundles to include for the final analysis. Minimum frequency scores vary significantly across studies, ranging from 40 times per million (e.g., Esfandiari and Barbary, 2017; Pan et al., 2016), to 25 times (Chen and Baker, 2010), to 20 times (Lu and Deng, 2019; Shirazizadeh and Amirfazlian, 2021). In terms of distribution across the corpus subparts, researchers have selected LBs occurring in a minimum of two texts (Wood and Appel, 2014), three texts (Chen and Baker, 2010), and five texts (Bychkovska and Lee, 2017). Other scholars, however, have opted for distribution cut-off thresholds based on specific proportion of at least 10% of all texts making up the corpus (Hyland, 2008b; Pérez-Llantada, 2014). Given that the two corpora are parallel in size, this study focuses LBs that recur at least 5 times. In addition, selected on LBs must be found in at least 8 lectures and five textbook chapters. This selection is based on 10% distribution threshold to ensure representativeness across both spoken and written data. The application of this proportional threshold accounts for the varying number of corpus sub-components, with the written data comprising 50 chapters and the spoken corpus consisting of 80 lectures. The list of the textbooks as well as the videotaped lectures is given in Appendix A.

### 3.3 Bundle identification and filtering procedures

AntConc Software (Anthony, 2024) is used to extract lexical bundles from the two corpora according to three major criteria: length of the target bundle, frequency of occurrence, and range across corpus sub-parts. This initial step has resulted in the extraction of a 404-item list of LBs from the spoken corpus and 116 from the written corpus. It seems clear that the corpus analysis may generate some LBs whose structural composition is exceedingly fragmentary. Examples include recurrent patterns such as *you do is you, you a little bit,* and *out to be a.* The deletion of such patterns is a common procedure in frequency-based studies of LBs (Hsu, 2014; Simpson-Vlach and Ellis, 2010). A total of 11 fragmentary bundles have been have been identified in the initial list obtained from the spoken register, whereas the list gleaned from the written corpus comprises two patterns. Excluding these fragmentary patterns has reduced the number of each list into 393 items in the spoken corpus and 115 in the written corpus (Appendices B, C).

### 3.4 Classifying bundles into distinct structural and functional categories

This study focuses on identifying LBs that are commonly used in two discourse registers related to the academic study of psychology. To draw a complete picture of LBs, it is important to classify them according to specific structural and functional categories. Three major grammatical categories are identified: patterns headed by noun phrases (NP-based), prepositions (PP-based), and verbs (VP-based). A small group of bundles does not neatly fall into any of these categories, highlighting the need for such unclassified bundles to be labeled as fragments (Cortes, 2004).

While classifying LBs into distinct grammatical groups is straightforward, the process of classifying bundles into functional groups is more complex. It involves using concordance lines to determine the target bundle’s function. The bundle, *as can be seen,* exemplifies disagreements between researchers regarding its functional category. Biber et al. (2004) believe that this bundle functions as a discourse marker, helping readers navigate the text. In contrast, Hyland (2008b) argues that *as can be seen* serves as a referential expression, alluding to visual information in the text.

This study adopts the functional framework created by Biber et al. (2004) because it accounts for bundles from both spoken and written corpora. According to this framework, lexical bundles are classified into three major categories: referentials, discourse organizers, and stance expressions. Subsequent studies have modified this framework, creating sub-categories to accommodate bundles that do not neatly fit within the three groups. In addition to referential expressions, discourse organizers, and stance signals, a fourth category was developed to account for expressions closely tied to the content of the specific domain under investigation (e.g., Breeze, 2013).

The log likelihood (LL) ratio is computed to assess the extent to which the functions and structures of lexical bundles differ between the two corpora. Raw frequencies of the target bundles are compared using Paul Rayson’s spreadsheet calculator, available at http://ucrel.lancs.ac.uk/llwizard.html. Lu and Deng (2019) reported LL values of 3.84, 6.63, 10.83, and 15.13, indicating *p*-values of <0.05, <0.01, <0.001, and <0.0001, respectively.

## 4 Findings

This study aims to investigate the use of lexical bundles (LBs) in two comparable psychology corpora. The first corpus comprises textbook chapters, representing the written register, while the second corpus includes academic lectures delivered at prominent academic institutions. Although the two corpora are closely aligned in their topical focus on psychology and the total number of running words, the analysis reveals that the spoken corpus contains substantially more LBs (types: 394, tokens: 9,936) than the written corpus of textbook chapters (types: 115, tokens: 2,064).

The high concentration of LBs in the spoken register aligns with earlier contrastive analyses, which indicate that oral academic language relies more heavily on recurrent formulaic patterns than its written counterpart (Biber, 2012; Nesi and Basturkmen, 2006).

### 4.1 Shared LBs

Since both registers focus on psychology, it is unsurprising that 31 lexical bundles (LBs) occur in both lists. The number of shared bundles represents 26.96% of all bundle types in the written register and 7.87% in the spoken register. In terms of tokens, shared bundles account for 35.91 and 9.78% of the total bundle occurrences in the written and spoken registers, respectively. Upon closer examination, six LBs contain discipline-specific node words around which other lexical items cluster (e.g., *of the brain is*, *parts of the brain*). Another subgroup consists of shared LBs that “occur regardless of their discipline, genre, or L1 background” (Esfandiari and Barbary, 2017, p. 30). Expressions such as *on the other hand*, *is one of the*, *one of the most*, and *as a result of* are reported in several studies (e.g., Ädel and Erman, 2012; Chen and Baker, 2010; Esfandiari and Barbary, 2017).

The distribution of these register-transcending LBs varies between the two corpora. Two-thirds of these shared bundles occur more frequently in the spoken register than in the written register, while one-third are more frequent in psychology writing than in lectures. Regarding their structural composition, the three major types are equally represented. A total of 11 LBs are verb-based, 10 are preposition-based, and nine are noun-based. The distribution of shared LBs functions across lectures and textbooks shows a strong preference for referentials which account for 67.7% of all instances. The prevalent presence of referentials suggests a more prominent role in presenting factual information by focusing attention on key topics, specifying details, framing discussion, indicating place references when necessary, and providing intangible framing to structure abstract concepts. Content-oriented group, which make up 19.4% of all shared LBs, indicate an emphasis on the conceptual and theoretical foundations of the subject matter. Stance (9.7%) and discourse-organizing (3.2%) bundles are relatively rare, likely due to their strict genre-specific usage, with stance bundles being more characteristic of the spoken discourse, while discourse organizers are commonly associated with the written texts. Given their presence in both spoken and written psychology discourse settings, these shared bundles can be pedagogically used to enhance the students’ ability to navigate both written and spoken discourse with greater fluency and precision. The following examples demonstrate the two shared bundles.

### 4.2 Structural classification

Table 2 provides the structural classification of LBs into three major types: NP-based, PP-based, and VP-based. A small subgroup does not align well with these categories, necessitating the creation of a fourth “fragment” category. There is noticeable variation in the distribution of LBs in the two corpora representing the study of psychology. Verb-based lexical bundles dominate academic lectures, accounting for nearly 80% of all types and tokens, while nominal and prepositional constructions feature more prominently in the written data. The dominant presence of phrasal structures and the relative scarcity of verb-based constructions in the written psychology corpus confirm findings from previous studies comparing speech and writing corpora for LBs.

**Table 2:** Structural classification of LBs.

Grammar category	Spoken corpus		Written corpus		LL	Example
	Types (%)	Tokens (%)	Types (%)	Tokens (%)		
*NP-based*	37 (9.39)	1,203 (12.11)	36 (31.3)	666 (32.28)	157.29^*^	*the size of the, the rest of the, one of the most, the central nervous system*
*PP-based*	32 (8.12)	784 (7.90)	37 (32.17)	760 (36.83)	0.41	*at the same time, in terms of the*, *in the same way, at the time of*
*VP-based*	312 (79.19)	7,727 (77.75)	36 (31.3)	554 (26.86)	7423.93^*^	*is part of the, are more likely to, play an important role, can be explained by*
*Fragments*	13 (3.3)	222 (2.24)	6 (5.23)	83 (4.03)	65.95^*^	*right in front of, than the sum of, the limbic system the*
Total	394 (100)	9,936 (100)	115 (100)	2,064 (100)		

^*^p < 0.0001.

Log-likelihood (LL) tests reveal three important results. First, psychology lecturers use NP- and VP-based bundle tokens significantly more often than psychology textbook authors. Second, LL tests indicate no significant differences between the two groups in the use of PP-based bundle tokens. Finally, the analysis reveals that fragmentary bundles are significantly more common in lectures than in writing. While the number of NP-based bundle types is nearly the same in both corpora, the overall number of NP-LB tokens is far greater in the spoken corpus than in the written corpus. This finding suggests that spoken registers are typically more repetitive and less innovative. The tendency to produce ill-formed, fragmentary structures in speech may be attributed to the situational characteristics of the speech genre, which, unlike written genres, lacks the opportunity for spaced revision and rephrasing.

### 4.3 Functional comparisons

The results shown in Table 3 demonstrate significant differences in the distribution of lexical bundles (LBs) between academic psychology lectures and psychology textbook chapters. In the spoken corpus, stance markers are the most prevalent functional category, accounting for nearly half of the bundle types and tokens. The considerable presence of stance markers in the spoken register is unsurprising, given the emphasis on the speaker’s perspectives. Referentials are the second most common functional category, reflecting their role in exemplifying ideas and concepts relevant to the study of psychology. Discourse organizers and content expressions are the least frequent groups.

**Table 3:** Functional classification of LBs.

Functions	Spoken corpus		Written corpus		LL
	Types (%)	Tokens (%)	Types (%)	Tokens (%)	
*Stance markers*	166 (42.13)	4,551 (45.80)	10 (8.70)	131 (6.35)	5301.97^*^
*Referentials*	140 (35.53)	3,228 (32.49)	67 (58.26)	1,280 (62.05)	873.07^*^
*Discourse organizers*	72 (18.27)	1,496 (15.06)	6 (5.22)	97 (4.70)	1479.55^*^
*Content*	16 (4.06)	661 (6.65)	32 (27.83)	555 (26.90)	9.41
Total	394 (100)	9,936 (100)	115 (100)	2,063 (100)	

^*^p < 0.0001.

In contrast, the written corpus of academic textbooks exhibits a different pattern, with referentials emerging as the most dominant functional category. This result suggests that the written psychology discourse is primarily concerned with disseminating core information rather than reflecting the author’s personal beliefs or subjective evaluations. Notably, stance markers and discourse organizers are much less common in the written corpus.

Overall, these findings highlight a marked contrast in the use of hedging functions between spoken and written academic discourse. The spoken corpus relies more heavily on stance markers, whereas the written corpus predominantly uses referentials. This suggests differing communicative strategies in spoken versus written academic contexts, potentially reflecting variations in interpersonal engagement and informational density.

Log-likelihood tests (Table 3) reveal significant differences in the distribution of the three major functional categories across the two registers. Lecturers use significantly more stance markers, referentials, and discourse organizers than textbook authors. However, no significant differences were found in the use of content LBs between the two groups. Consistent with these findings, stance markers are more prominent in the spoken corpus of psychology lectures, whereas referentials are more frequently used in psychology textbook chapters.

While these results align with the findings of Liu and Chen (2020) regarding the infrequent use of discourse organizers in academic lectures, they differ markedly in the use of referentials. Unlike stance markers, referentials are ranked as the most dominant functional category in this study.

#### 4.3.1 Academic psychology texts

Within each register, the functional distribution of bundles varies significantly. In the following sections, I have a close examination of the sub-categories of the three major functions in the written psychology corpus.

#### 4.3.2 Referential expressions

**Figure 1:**
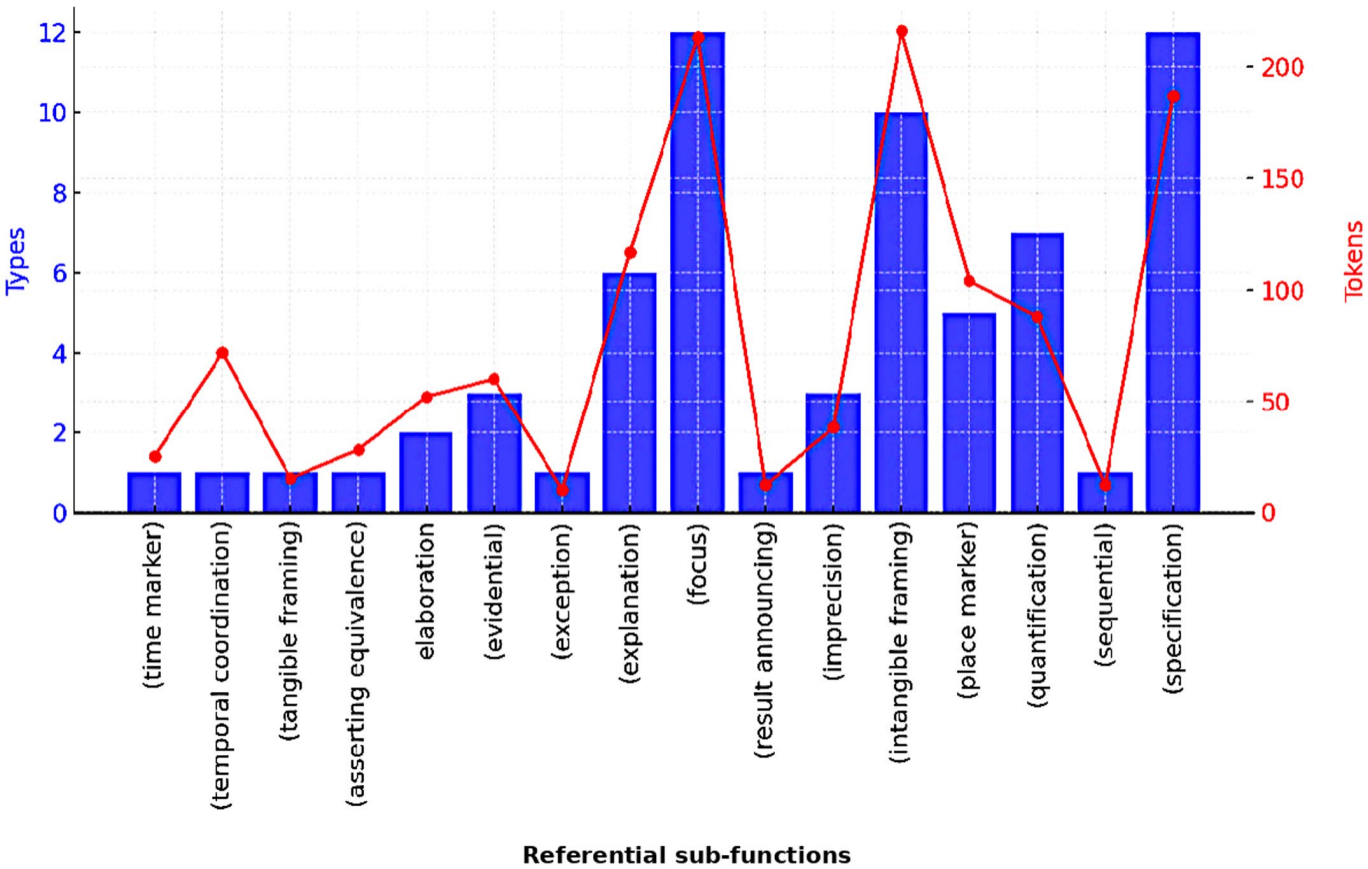
Referential sub-functions in textbooks.

Referential bundles are employed to “identify an entity or to emphasize certain aspects of an entity as especially important.” (Ren, 2021, p. 7). These bundles can be divided into several sub-groups, including focus, specification, and intangible framing. Bundles used to fulfill these sub-groups account for nearly half of the bundle types and tokens in the referential category (Table 4). As illustrated in Figure 1, some referential sub-categories tend to have a relatively lower number of bundle types; yet, their total frequency counts (tokens) are considerably higher. The following examples illustrate the meaning of two referential bundles: *at the same time* and *one of the most.*

- “People with Wernicke’s aphasia have a general impairment of language comprehension, while *at the same time* speech production is intact.”
- “We also know that *one of the most* common responses to frustration is aggression.”

**Table 4:** Referentials in psychology textbooks.

Referential sub-functions	type	%	token	%
Time marker	1	1.49	25	2.00
Temporal coordination	1	1.49	72	5.76
Tangible framing	1	1.49	15	1.20
Asserting equivalence	1	1.49	28	2.24
Elaboration	2	2.99	52	4.16
Evidential	3	4.48	60	4.80
Exception	1	1.49	10	0.80
Explanation	6	8.96	117	9.37
Focus	12	17.91	213	17.06
Result announcing	1	1.49	12	0.96
Imprecision	3	4.48	38	3.04
Intangible framing	10	14.93	216	17.29
Place marker	5	7.46	104	8.33
Quantification	7	10.45	88	7.05
Sequential	1	1.49	12	0.96
Specification	12	17.91	187	14.98
Total	67	(100)	1,249	(100)

Figure 1 demonstrates that some sub-groups such as *temporal coordination* and *intangible framing* show high token counts, but exhibit rather limited diversity. The repeated use of patterns such as *in the form of, in terms of the, on the basis of,* and *in the presence of* confirm their utility for content construction.

#### 4.3.3 Content expressions

The second-largest functional category of LBs comprises expressions closely related to the study of psychology as an academic discipline. In most cases, the lexical bundle consists of content words accompanied by one or two non-content items (e.g., the central nervous system, in the left hemisphere). Examples from the written corpus, where LBs are primarily composed of content words, include:

- “*The central nervous system* in vertebrates separates sensory and motor processing.”
- “Functional magnetic resonance imaging (fMRI) confirms that sensory and motor areas *of the brain* are more active when a person feels a phantom limb.”

The prevalent occurrence of content words within the written psychology corpus is unsurprising, given the situational characteristics and communicative purposes of textbooks, which aim to disseminate specialized disciplinary knowledge (Biber and Conrad, 2009). A notable observation regarding the grammatical structure of LBs is that 25 out of the 32 identified content expressions consist of complex noun-phrase constructions.

#### 4.3.4 Stance expressions

The third functional group within the written psychology corpus comprises bundles performing a stance function. Stance expressions are broadly defined as patterns “used either to evaluate the status of knowledge or to state ability/desire to achieve certain results” (Ren, 2021, p. 8). Four subcategories are identified: ability, epistemic perspective, possibility, and reader engagement. Expressions representing possibility and epistemic perspectives account for two-thirds of all stance markers, while ability and reader engagement are the least frequent, with fewer types. When tokens are counted, epistemic and possibility patterns constitute 85% of all stance bundle tokens. The limited use of stance bundles in the written register has been interpreted from a register perspective, as “textbook language is commonly packaged as simple factual reporting of information, a faceless stance with no indication of personal attitude” (Biber, 2006, p. 113). The following examples demonstrate instances of stance bundles:

1. “Explain why *it is important to* measure brain function at the basic level of neuronal activity.”
2. “They want *to be able to* explain these relationships, too.”

Table 5 presents stance expressions in psychology textbooks categorized by sub-functions.

**Table 5:** Stance expressions in psychology textbooks.

Stance sub-functions	Types	%	Tokens	%
Ability	2	18.18	21	12.35
Epistemic	4	36.36	58	34.12
Possibility	4	36.36	84	49.41
Reader engagement	1	9.09	7	4.12
Total	11	(100)	170	(100%)

**Figure 2:**
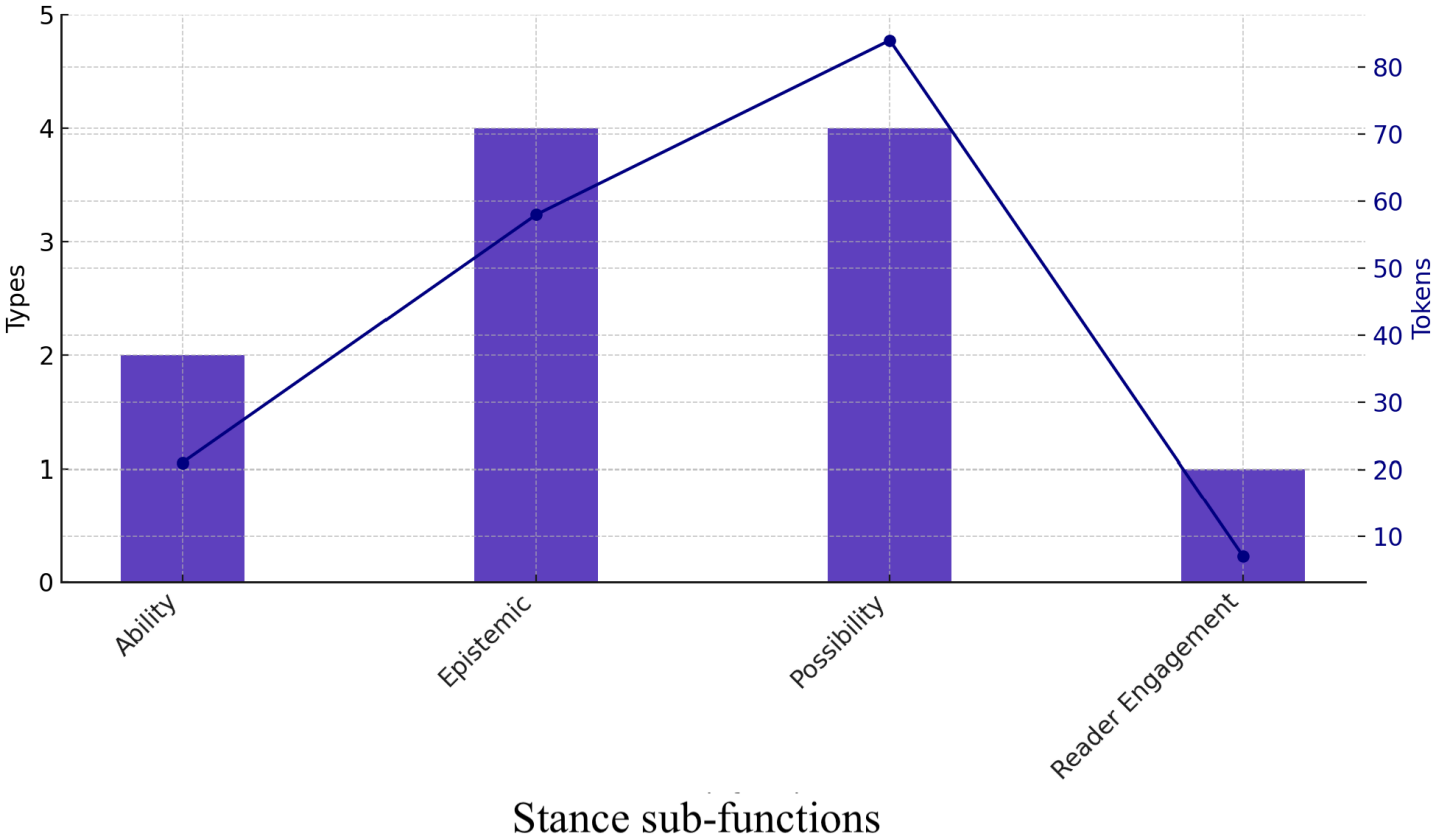
Stance sub-functions in textbooks.

Figure 2 reveals that bundles expressing possibility occur far more frequently than epistemic bundles although the two sub-groups have an equivalent number of bundle types.

#### 4.3.5 Discourse organizers

Discourse organizers, the least commonly used functional category in the written register, can be classified into five distinct sub-groups: indicating causal relationships (e.g., the effects of the), providing additional information (e.g., as well as the), marking contrastive relations (e.g., on the other hand), and referring to spatial and temporal placement (e.g., at the beginning of). The examples below highlight some discourse organizers uncovered by the analysis:

1. “Two major problems in naturalistic observation are *the effects of the* observer and observer bias.”
2. “Be sure to describe feelings *as well as the* plot, characters, and actions of the dream.”

Table 6 classified the distribution of discourse organizers in psychology textbooks by their sub-functions.

**Table 6:** Discourse organizers in the psychology textbooks.

Discourse organizing sub-functions	Types	%	Tokens	%
Causal relationship	2	40	26	29.21
Additional	1	20	8	8.99
Contrastive marker	1	20	40	44.94
Cross reference	1	20	15	16.85
Total	5	(100)	89	(100)

**Figure 3:**
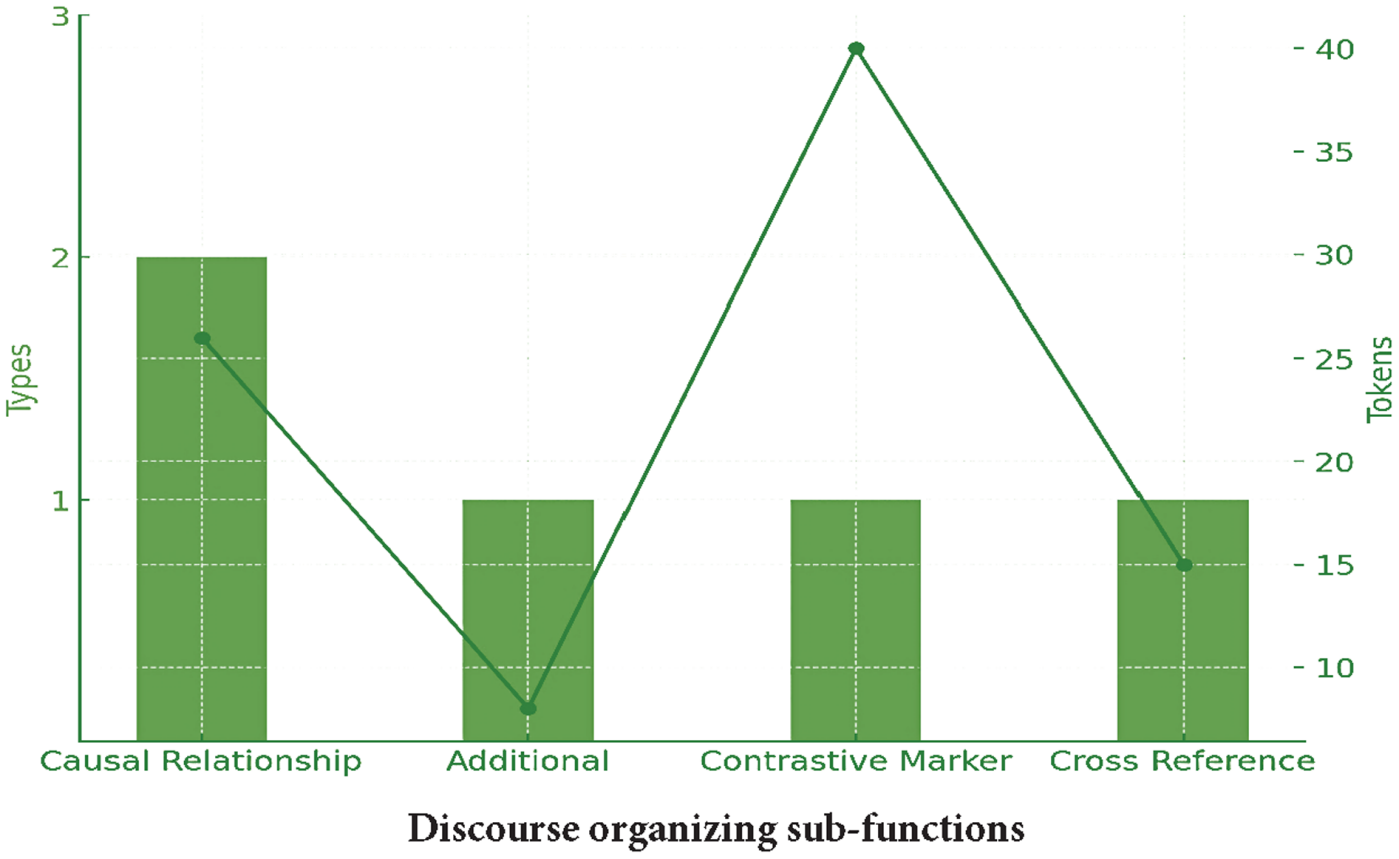
Discourse-organizing sub-functions in textbooks.

Figure 3 illustrates the distribution of discourse organizers in the written psychology corpus, showing that while bundle types remain relatively consistent across the four sub-functions, their overall occurrences vary significantly, with the contrastive marker recurring more frequently than other sub-groups.

### 4.4 Academic psychology lectures

The spoken psychology register, in contrast to the written psychology register, exhibits significantly higher number of lexical bundle types and tokens. A functional classification reveals that most LBs serve a stance function, while the second most prevalent category comprises LBs that perform a referential function. Discourse-organizing bundles and content expressions are ranked third and fourth functional groups, respectively. The dominant presence of stance markers in the spoken register appears to be congruent with previous research findings analyzing LBs in speech registers. Biber and Barbieri (2007) indicate that stance “is a general characteristic of spoken university register” (p. 274). Discourse-organizing expressions are the least frequently occurring functional category, a pattern consistently observed in studies on spoken registers (e.g., Wang and Csomay, 2024). Csomay (2013) further notes that as the classroom discussion progresses, the reliance on discourse organizers diminishes. The need for more discourse organizers diminishes. Unlike discourse organizers, however, referentials and stance markers remain essential throughout instructional sessions, facilitating more focused communication and interactions.

#### 4.4.1 Content bundles

Eight LBs, occurring 506 times, are identified as containing technical terms closely associated with the field of psychology. The discipline-specific nature of some LBs is highlighted in prior research on LBs (e.g., Cortes, 2004; Hyland, 2008a; Jablonkai, 2010; Qin, 2014). Given that psychology focuses on mental processes and behaviors governed by the brain, it is unsurprising to find that many LBs include the term *brain* either bundle-initially (e.g., the brain is the) or bundle-finally (e.g., part of the brain). Several other content LBs describe the composition of the human brain such as *the central nervous system* and *areas of the brain*. A comparison involving these discipline-specific LBs across the two corpora reveals no statistically significant differences, suggesting their consistent use regardless of the specific corpus under analysis. Below are examples that highlight some content bundles used in the spoken corpus:

1. “We really test a part of *the brain that is* there.”
2. “And to reduce its representation of *parts of the body* that it does not have to do too much with.”

Table 7 provides the list of content-oriented LBs in the spoken register.

**Table 7:** Content LBs in the psychology lectures.

Function	Bundle	Freq.
*Content*	1. Part of the brain	216
	2. Of the brain that	98
	3. Parts of the brain	89
	4. Of the brain and	56
	5. The brain and the	23
	6. The brain is the	8
	7. The brain that is	8
	8. What the brain does	8
Total	Types (8)	Tokens (506)

#### 4.4.2 Stance bundles

The majority of LBs in the spoken register fulfill a stance function, highlighting the essential role of this functional category in shaping and disseminating the oral discourse. The frequent use of stance bundles necessitates their classification into sub-categories, reflecting the diversity of linguistic patterns and the communicative strategies employed to convey stance. As shown in Table 8, the analysis identifies several distinct subcategories, each performing a specific sub-function. These subcategories include LBs that express the speaker’s intent (e.g., *I am going to*), prediction (e.g., it is going to be), listener engagement (e.g., you do not have), desire (e.g., you want to know), and epistemic knowledge (e.g., you do not know). This classification accounts for the complexity of stance bundles as they accommodate both the speaker’s objectives and the audience’s expectations.

1. “And *I am going to* ask actually for a vote because I’m going to return to this.”
2. “Oh my god! *What is going on* here? This is pitiful.”

Table 8 presents a detailed analysis of stance sub-functions, highlighting their distribution across types and tokens.

**Table 8:** Stance sub-functions in psychology lectures.

Stance sub-function	Types	%	Tokens	%
*Intention*	12	16.00	1,153	36.04
*Prediction*	4	5.33	208	6.50
*Listener engagement*	8	10.67	297	9.28
*Desire*	9	12.00	275	8.60
*Epistemic*	8	10.67	209	6.53
*Lack of desire*	6	8.00	189	5.91
*Unexpected outcome*	5	6.67	156	4.88
*Evaluation and judgment*	9	12.00	288	9.00
*Uncertainty*	4	5.33	133	4.16
*Unnecessity*	2	2.67	71	2.22
*Lack of understanding*	4	5.33	119	3.72
*Ability*	2	2.67	45	1.41
*Emphasis*	2	2.67	56	1.75
Total	*75*	(100)	3,199	(100)

**Figure 4:**
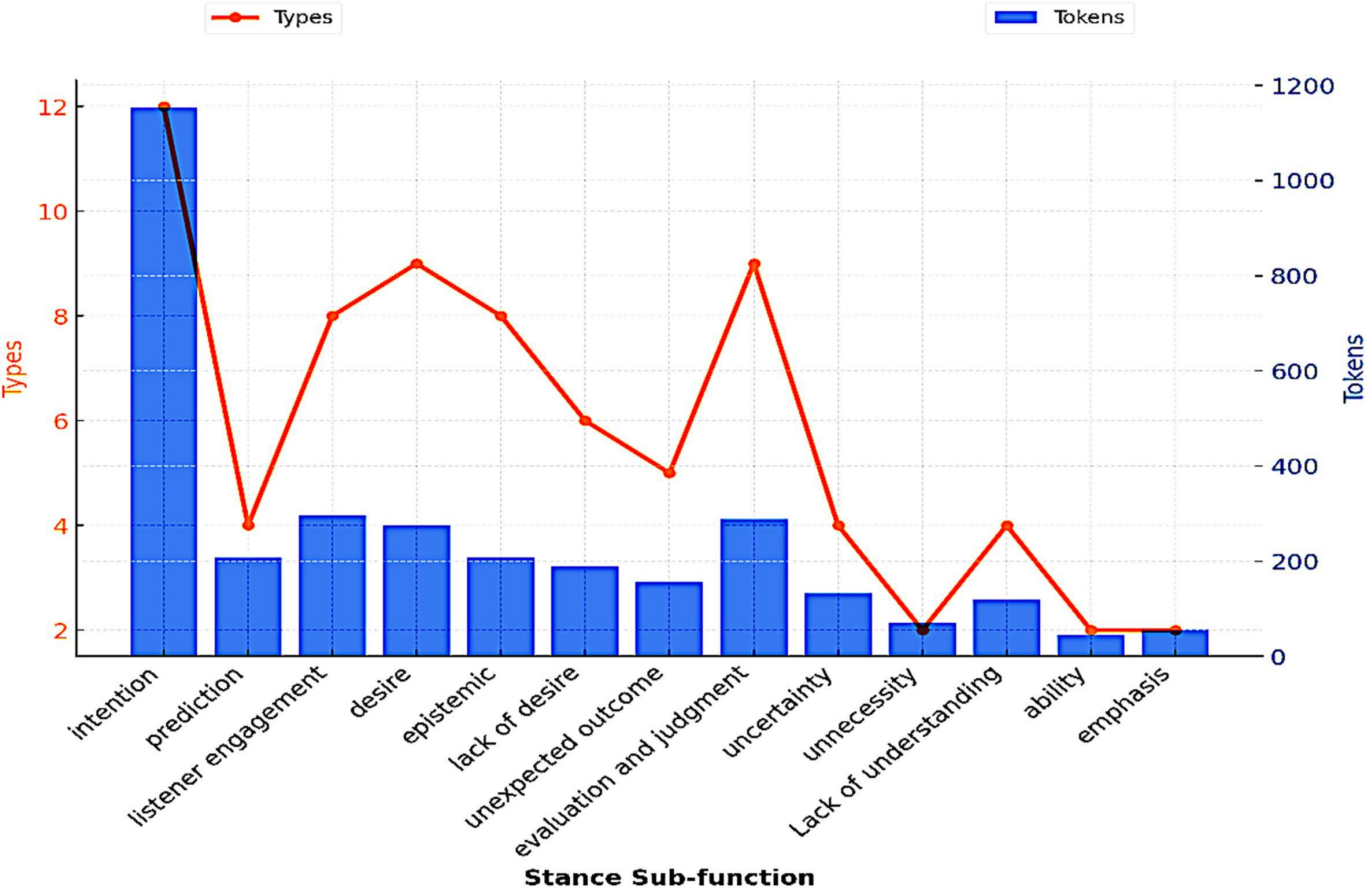
Stance sub-functions in lectures.

Within this subcategory, Figure 4 demonstrates that academic lectures rely on a limited set of expressions, which are repeatedly utilized to convey stance. The repeated use of the same stance patterns while speaking may be interpreted as a strategy to meet the cognitive demands of the real-time communication.

#### 4.4.3 Referential bundles

The second most prominent group within the spoken corpus consists of bundles fulfilling a referential function. This group can be further categorized into four key sub-functions: focus, quantification, specification, and repetition. Bundles associated with the focus sub-function exhibit a total of 10 distinct types, collectively occurring 382 times. In contrast, bundles performing a quantification sub-function, though limited to just seven types, display a higher frequency profile, appearing 406 times across the corpus. Here are two examples showing bundles which perform a referential function:

1. “Talk *a little bit about* the enterprise of trying to say which parts of our brain support which parts of our mind.”
2. “We’ll talk *a little bit about* patients with Prosopagnosia.”

Table 9 summarizes the distribution of referential bundles within the spoken psychology corpus.

**Table 9:** Referentials sub-functions in psychology lectures.

Referential sub-function	Types	%	Tokens	%
*Focus*	10	37.04	382	33.75
*Quantification*	7	25.93	406	35.87
*Specification*	7	25.93	259	22.88
*Repetition*	3	11.11	85	7.51
Total	27	(100)	1,132	(100)

**Figure 5:**
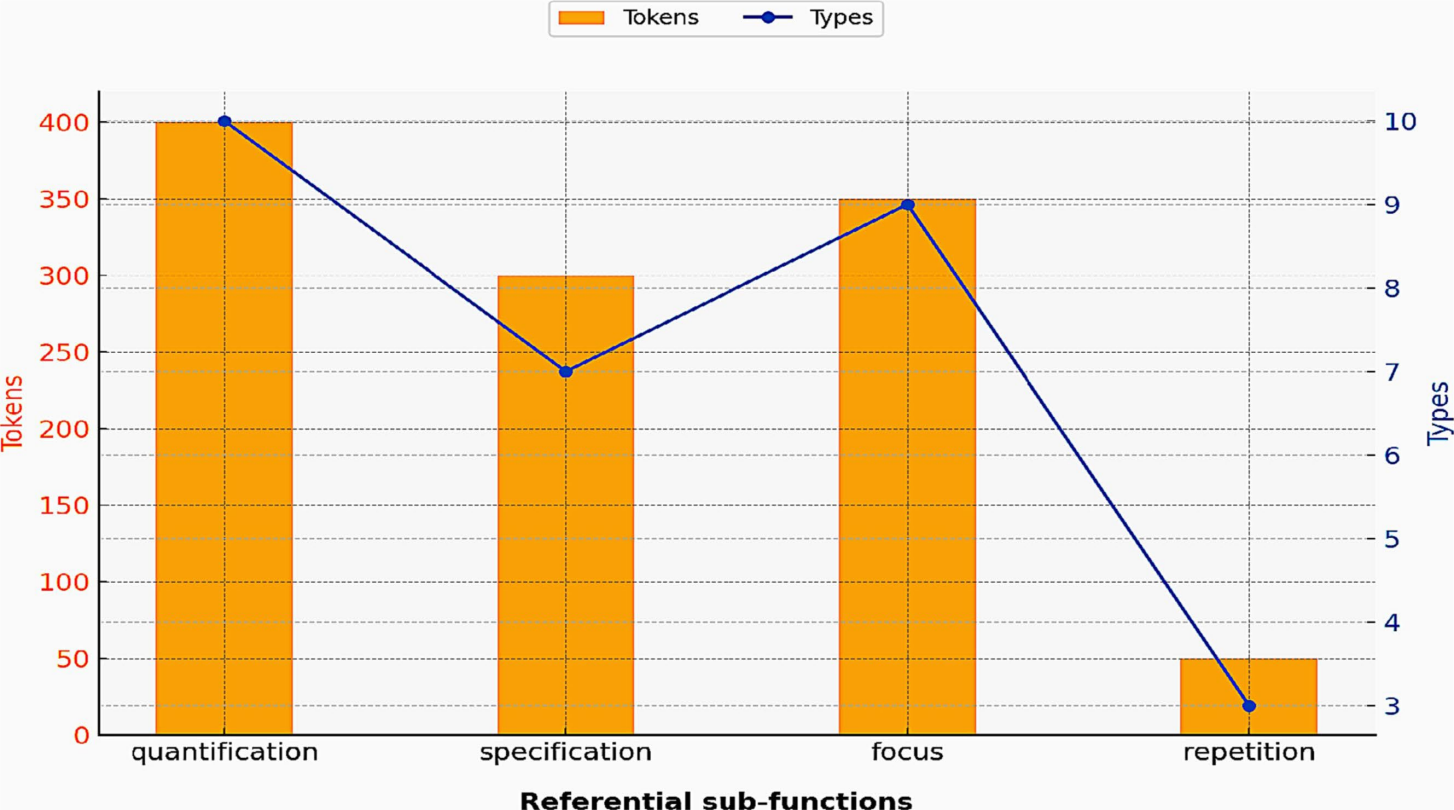
Referential sub-functions in lectures.

Figure 5 shows that sub-groups with a higher number of types, such as quantification and focus, tend to recur more frequently, as is evidently clear from their higher token counts. In contrast, sub-groups such as specification and repetition exhibit a correspondingly lower token counts, indicating their less robust presence.

#### 4.4.4 Discourse organizers

**Figure 6:**
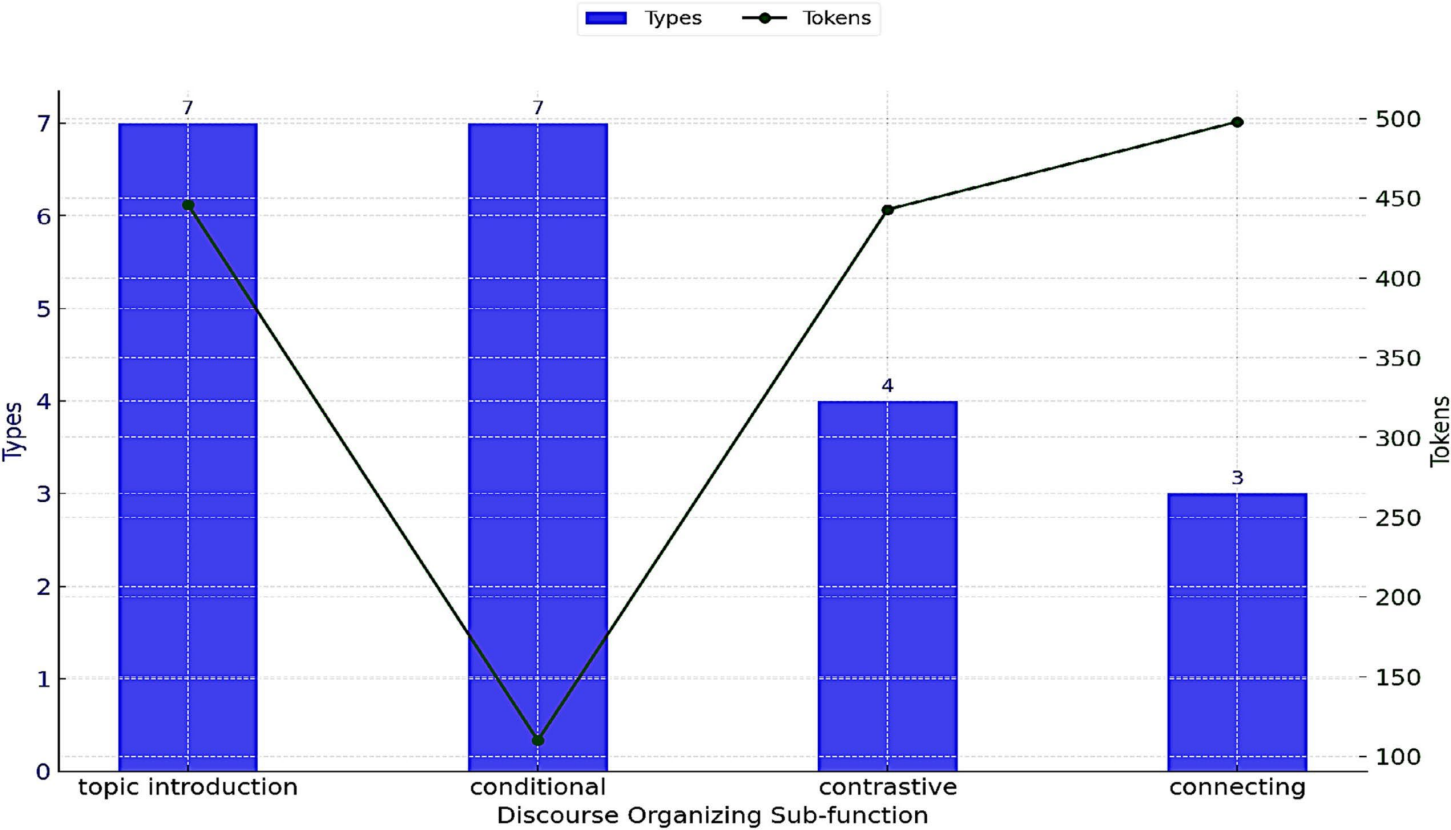
Discourse-organizing sub-functions in lectures.

The third functional category identified within the spoken corpus includes discourse-organizing bundles (Table 10). These bundles serve to introduce a topic, establish conditional and contrastive relationships, and help to link two parts of the discourse. Although this category ranks third in terms of the diversity of patterns, discourse organizers outnumber referential bundles in the overall frequency counts. Moreover, substantial variation exists within the sub-functions of these discourse organizers, as patterns functioning to connect parts of the discourse occur more frequently, though are represented by few distinct bundle types. Figure 6 illustrates the LBs performing a discourse-organizing function, categorized into four sub-groups. Though limited to few types, bundles serving to connect discourse and to establish contrastive relations have the highest token counts. The following examples show the actual use of two discourse organizers: *if you have a* and *what you do is*.

1. “Outlets for frustration, *if you have a* good outlet for frustration, that diminishes the toxic effects of stress.”
2. “And, as a female hamster, *what you do is* you ovulate every five days or so.”

**Table 10:** Discourse organizers sub-functions in psychology lectures.

Discourse organizing sub-function	Types	%	Tokens	%
*Topic introduction*	7	33.33	446	29.79
*Conditional*	7	33.33	110	7.35
*Contrastive*	4	19.05	443	29.59
*Connecting*	3	14.29	498	33.27
Total	21	(100)	1,497	(100)

## 5 Discussion

Corpus linguistics plays an important role in understanding linguistic variation across different disciplines, registers, or genres (Biber et al., 1999). Through the analytical lens of corpus linguistics, large collections of authentic language data can be analyzed for patterns of use either across or within different contexts. Research in corpus linguistics has shown that speakers and writers draw on prefabricated chuncks to enhance fluency and facilitate rapid cognitive processing, an observation that can now be empirically substantiated. The prevalent use of prefabricated chuncks has been conceptually studied under the broader notion of formulaic language, an umbrella term that includes other related concepts such as lexical bundles (Wray, 2002). Sinclair (1991) framed this linguistic phenomenon within the term “idiom principle,” suggesting that language users resort to ready-made chunks while speaking or writing and that these chunks can be obtained, analyzed, and interpreted using a combination of corpus methodology and linguistic analysis.

Building on these theoretical considerations, this study contrastively analyzes the presence of four-word lexical bundles (LBs) in two corpora representing spoken and written psychology registers: academic lectures and textbook chapters. Controlling for factors such as corpus size, disciplinary focus, frequency, and distribution, the study identified 394 types and 9,936 tokens in the spoken psychology corpus, compared to 115 types and 2,063 tokens in the written corpus. These findings demonstrate that LBs are sensitive to register differences (Biber, 2012; Biber and Gray, 2010), with the spoken psychology exhibiting a significantly wider variety of LBs. Conversely, the written psychology register contains a limited number of bundle types and tokens. This variation suggests that neglecting register differences in describing and evaluating linguistic patterns may lead to inaccurate generalizations and incomplete understanding of language use within psychology as an academic discipline. The disparity between the two registers seems to be consistent with the results of previous comparative analyses, which show that spoken registers rely on a far greater number of LBs than written academic registers (Biber and Barbieri, 2007; Hoang and Crosthwaite, 2024). Biber and Barbieri (2007) noted that “Lexical bundles are generally rare in textbooks and academic prose” (p. 278). Biber et al. (2004) attributes this rarity to the distinct situational characteristics of the registers. Unlike face-to-face instruction, writing a textbook chapter involves far less time pressure, enabling authors to explore different lexical options and continually refine their language for clarity, precision, and alignment with informational focus on the subject matter. Another explanation relates to the communicative purpose of academic textbooks, which serve as means for disseminating subject-specific content. Hyland (2009) points out that textbooks authors “draw on the genres, models, and beliefs of their communities in constructing their material, representing their field in particular ways.” (p. 113). This emphasis on discipline-specific representation makes textbook language more elaborate and less repetitive than the language used in academic lectures.

Bundles typical of the “literate” register are structurally and functionally different from LBs used in the “oral” register (Nesi and Basturkmen, 2006). A closer examination of the top five LBs in the written corpus (*at the same time, the central nervous system, parts of the brain, one of the most, on the other hand*) reveals that they are structurally nominal or prepositional, and primarily serve a content or referential function. By contrast, the top LBs in the list derived from the spoken corpus (*I am going to, we are going to, part of the brain, you are going to, what is going on*), show a distinct shift toward verb-based, stance-functioning patterns. The dominant presence of these predictive patterns in the spoken register reflects the dynamic and interactive nature of the spoken classroom discussions (Biber and Barbieri, 2007).

Turning to the structural characteristics of patterns, the majority of LBs in the written psychology register are phrase-based, whereas LBs in the spoken register are mostly verb-based. The concentration of phrasal constructions of LBs in the written academic register is consistent with several previous findings (Biber, 2006; Biber et al., 2004; Biber and Barbieri, 2007; Chen and Baker, 2010; Cortes, 2004; Esfandiari and Barbary, 2017; Hyland, 2008a; Jablonkai, 2010). Some researchers interpret the extensive use of phrasal constructions in the academic prose by the need for “informational focus” (Pan et al., 2016) in which meaning relations can be adequately fulfilled by an extensive use of non-clausal expressions. Relying more on nominal/phrasal constructions in academic texts is also seen as a feature of academic prose which, unlike conversations, is “structurally compressed rather than elaborated” (Biber and Gray, 2010, p. 5).

Consistent with previous research analyses, LBs emerging from both corpora have been investigated in terms of their functional characteristics, resulting in the identification of three major categories: referentials, stance markers, and discourse-organizers. It is clear that referentials are dominant in the textbook register, while stance markers are prioritized in the psychology lectures. The less use of stance bundles in the written corpus can be interpreted from a register perspective, as “textbook language is commonly packaged as a simple factual reporting of information, a faceless stance with no indication of personal attitude” (Biber, 2006, p. 113). Hyland (2008a, 2008b) maintained that the academic prose, including textbooks, tends to make a greater use of referentials at the expense of stance markers and discourse organizers. In textbooks, furthermore, the tendency to rely less on stance markers is explained as a “way to promote the objectivity and to exclude personal interest in a claim” (Ren, 2021, p. 9). In such case, “factual information” is delivered “with no overt marking of stance” (Biber, 2006, p. 114).

Some researchers have described certain LBs as “semantically transparent and syntactically flexible” (Wang, 2019, p. 60). However, a detailed analysis of concordance lines indicates that LBs do not follow a consistent pattern across different contexts. For instance, the meaning of the expression *in terms of the* cannot be determined by identifying the meaning of the single expression *terms,* as can be exemplified in the following examples:

1. *Evolutionary psychologists try to explain human behavior in terms of the underlying computations that occur within the mind.*
2. *In terms of the distinction we encountered earlier, remembering is associated with the episodic memory.*

These deceptively transparent expressions (Martinez and Schmitt, 2012) may go unnoticed by learners of English and materials designers, as these patterns often appear to pose no significant challenge. However, caution must be exercised before categorizing LBs as semantically transparent/non-transparent without examining the behavior of these patterns in multiple contexts. A small proportion of patterns identified in the two corpora are discipline-dependent (Ren, 2021), or content-based (Breeze, 2013). They are functionally referential and structurally phrasal and tend to occur in the textbook chapters more than in academic lectures.

This study extends prior research which focuses on LBs within distinct academic domains, including history and biology (Cortes, 2004), linguistics (Shirazizadeh and Amirfazlian, 2021) engineering (Wood and Appel, 2014) pharmacy (Grabowski, 2015), mathematics (Alasmary, 2019), and law (Breeze, 2013). A common thread across all these studies is the classification of LBs into distinct structural and functions groups. Unlike previous research, this study unveils several functional sub-groups that are not accounted for either in register or discipline-based studies. Referentials, for example, can be grouped into several functional sub-categories, some of which have not been accounted for by previous classification schemes. A bundle such as *it is the same as* is a referential used to mark equivalence, whereas *for those of you* is a discourse organizing expression employed to specify sub-group to which an individual belongs. Furthermore, this study has uncovered several instances of LBs which are deeply tied to psychology as an academic discipline, with far lower chance of being encountered across other disciplines. Examples include sequences such as *the central nervous system, parts of the brain,* and *areas of the brain.* These LBs are shaped by the linguistic choices and rhetorical conventions within psychology.

In conclusion, it is important to highlight three major contributions of this study. First, the contrastive analysis of the bundles within the two registers confirms a gap observed in previous research studies, showing that the spoken academic register includes a broader range of LBs than the written academic register. This gap has not been empirically substantiated within psychology, highlighting the need to determine whether similar patterns hold across other academic domains. Second, the study highlights the nuanced structural and functional distinctions of LBs in psychology, emphasizing the need for pedagogical interventions to address the lexical demands, both spoken and written, of an academic study. A third important contribution lies in the identification of several key sub-functions that these LBs perform in the psychology discourse. Previous functional classification frameworks of LBs into referentials, stance signals, and discourse organizers should serve as a primary step, followed by a close examination of concordance lines in order to uncover the full range of additional of sub-functions that are not accounted for in prior research due to register or discipline variation.

## 6 Pedagogical implications and limitation

Through a combination of corpus treatment and linguistic analysis, the current study has examined LBs in two underexplored registers in the discipline of psychology. The list of LBs is hoped to inform future research and shape practice in psychology education. LBs can be introduced across several contexts so as to aid noticing and maximize exposure. Northbrook and Conklin (2019) demonstrated that “Beginners are sensitive to the frequency of lexical patterns in their input, which highlights the importance of presenting learners with authentic formulaic language in their textbooks.” (p. 12). Items on the two lists can be used for a wide range of pedagogical purposes. For example, they may be used to alert psychology students to genre distinctions (Biber, 2012; Shin and Won, 2024), draw attention to proper grammatical choices (Kang et al., 2024; Shin et al., 2018), and pinpoint adequate rhetorical moves (Cortes, 2013; Farhang-Ju et al., 2024). In addition, LBs gleaned from both the written corpus (Appendix B) and the spoken corpus (Appendix C) can be explicitly used as part of an English for Specific/Academic Purposes program to enhance academic writing, classroom discussions, presentations, and scholarly debates. Bundles such as *come up with the* and *turns out to be* can be contextualized using a concordance tool, thus preparing psychology students to initiate and sustain classroom communication. For psychology writing, instructors may integrate some LBs into a task-based activity, helping them apply newly acquired knowledge in writing term papers or responding to short, information-seeking prompts. Extensive exposure to authentic language data is expected to support learners in transitioning from novice writing styles to more expert-like ones.

While this study provides a foundation for future research involving cross-corpora comparisons of different psychology registers, it is important to acknowledge three important limitations. First, the number of types and tokens in this study is generated using arbitrarily established criteria of length, frequency, and dispersion. Samraj (2024) pointed out that applying different criteria can influence the results, generating different number of bundle types and tokens. Second, the current research is confined to academic lectures and textbook chapters. Expanding the scope of research to include other registers such as counselor-patient discussions, journal articles, and dissertations/theses could have offered a more comprehensive view of the types and functions of LBs in other psychology-related contexts. A third limitation concerns the generalizability of the findings gleaned from discipline-specific, register-focused study, restricting its applicability to other fields with distinct rhetorical conventions, communicative purposes, and situational characteristics. Nevertheless, this study has shed light on the use of LBs within the scope of academic lectures and textbooks with far greater implications for psychology learning and writing.

## 7 Conclusion

Previous corpus-based studies have explored the use of lexical bundles in a wide range of disciplines, genres, and registers. The current study extends this line of inquiry by examining lexical bundles in two spoken and written psychology registers. This result is consistent with previously reported conclusions that speech is inherently formulaic, routinized, and more prefabricated than writing. Although the two corpora are topically focused on psychology as an academic discipline, yet each corpus seems to prioritize certain LBs. The analysis further revealed such distinct characteristics exhibited by the two registers also influence the functions and the structural forms of LBs, with referentials featuring more prominently in the written psychology chapters, whereas stance expressions are more ubiquitous of LBs in lectures. These findings suggest that intradisciplinary variation is as prevalent as interdisciplinary variation and that English for Specific/Academic Purposes programs should be aware of the distinct needs of individuals while designing and implementing courses and programs even within closely related genres and registers. As Esfandiari and Barbary (2017, p. 11) put it, “Association and dissociation of certain linguistic features with given disciplines, however, run the risk of misleading EAP practitioners and students by making them think that they should use certain features and avoid others in all their disciplinary writing regardless of genre and text sections.”

To conclude, it is important for scholars with an interest in lexical bundles to extend the line of their inquiry beyond the study of conformity or variation across registers. By exploring a broader spectrum of other linguistic patterns, functions, and structural forms, researchers can obtain a deeper understanding of how disciplinary communities use language to create, disseminate and interpret content. Such approach can enrich pedagogical practices, foster better disciplinary writing, and develop more comprehensive knowledge of academic language.

## Data Availability

The original contributions presented in the study are included in the article/supplementary material, further inquiries can be directed to the corresponding author.
