# Supplementary material for: Lexical bundles in psychology lectures and textbooks: a contrastive corpus-based study with implications for academic writing
Source: Front Psychol. 2025 Apr 14;16:1545355. doi: 10.3389/fpsyg.2025.1545355 (PMC12034667; doi:10.3389/fpsyg.2025.1545355)
Supplement: Supplementary file 1 [file Data_Sheet_1.pdf]

Supplementary materials

Appendix (A)

First: The textbooks

Weiten, W. (2014). *Psychology: Themes and variations* (9th ed.). Cengage Learning.

Gross, R. (2015). *Psychology: The science of mind and behavior* (7th ed.). Hodder Education.

Schacter, D., Gilbert, D., Wegner, D., & Hood, B. (2014). *Psychology* (4th ed.). Palgrave Macmillan.

Popov, A., Parker, L., & Seath, D. (2017). *Psychology*. Oxford University Press.

Coon, D., & Mitterer, J. O. (2013). *Introduction to psychology: Gateways to mind and behavior* (13th ed.). Wadsworth.

First: The videotaped lectures

1. Yale courses

	Course title	*Duration (H:M)*
6.	Introduction	00:29:
7.	Foundations: This is your brain	00:53:
8.	Foundations: Freud	00:56
9.	Foundations: Skinner	00:58
10.	The development of thought	00:48
11.	Language in the brain, mouth	00:56
12.	Consciousness of the present: consciousness of the past: language	00:59
13.	Consciousness of the present; consciousness of the past	00:59
14.	Evolution, emotion, and reason: Evolution and Rationality	00:59

15.	Evolution, emotion, and reason: Emotions Part I	00:53
16.	Evolution, emotion, and reason: Emotions Part II	00:56
17.	Evolution, emotion, and reason: Emotions Part III	01:05
18.	Why are people different? Differences	01:05
19.	What motivates us: Sex.	00:52
20.	A person in the world of people: morality .15	01:01
21.	A person in the world of people: self and other 16 Part I	00:58
22.	A person in the world of people: self and other, part II	01:04
23.	What happens when things go wrong: mental illness. Part I	00:54
24.	What happens when things go wrong: mental illness. Part II	00:56
25.	The good life: Happiness	00:47

2. MIT courses

Course title	Duration (h:m)
1. Introduction to psychology: Lecture 1	00:49:
2. Introduction to psychology: lecture 2	01:11
3. Introduction to psychology: Lecture 3	00:54
4. Introduction to psychology: Lecture 4	01:11
5. Introduction to psychology: Lecture 5	00:40
6. Introduction to psychology: Lecture 6	00:56

7. Introduction to psychology: Lecture 7	00:42
8. Introduction	01:19
9. Neuroanatomy	00:50
10. Cognitive neuroscience methods I	01:00
11. Cognitive Neuroscience methods II	01:11
12. Experimental design	00:56
13. Category selectivity, controversies, and MVPA	01:09
14. Navigation I	01:23
15. Navigation II	01:11
16. Development, Nature, & Nurture I	01:21
17. Development, Nature, & Nurture II	01:17
18. Number	01:10
19. Hearing & speech	01: 18
20. Music	01:12
21. Language I	01:08
22. Mentalizing and Theory of Mind	01:16
23. Brain networks	01:23
24. Attention and awareness	00:56

3. Sandford

Course title	Duration (h:m)
1. Introduction to human behavioral biology	00:57
2. Behvioral evolution	01:36
3. Behavioral evolution II	01:36

4. Molecular Genetics I	01:33
5. Molecular Genetics II	01:14
6. Behavioral Genetics I	01:38
7. Behavioral Genetics II	01:32
8. Recognizing relatives	01:19
9. Ethology	01:41
10. Introduction to Neuroscience I	01:00
11. Introduction to Neuroscience II	01:13
12. Endocrinology	00:49
13. Advanced Neurology and Endocrinology	01:13
14. Limbic System	01:28
15. Human sextual behavior I	01:40
16. Human sextual behavior II	01:40
17. Human sextual behavior III & aggression I	01:36
18. Aggression II	01: 45
19. Aggression III	01:41
20. Agression IV	01:42
21. Chaos and reductionism	01:37
22. Emergence & complexity	01:42
23. Language	01:42
24. Individual differences	00:53

4. Berkeley

Course title	*Duration (H:M)*

1. Lecture 1: general psychology	00:48
2. Lecture 2: biological bases of mind and behavior	00:40
3. Lecture 3: biological bases of mind and behavior	00:50
4. Lecture 4: biological bases of mind and behvior	00:48
5. Lecture 5: Learning 1-general psychology	00:49
6. Lecture 6: learning 2-general psychology	00:48
7. Lecture 7: learning 3-general psychology	00:50
8. Lecture 8: sensation and perception 1-general psychology	00:49
9. Lecture 9: sensation and perception 2-general psychology	00:49

Appendix (B)

No	Lexical bundle	Freq	Sub-functions	Grammar structure
1.	**at the same time**[1]	72	Referential (temporal coordination)	Preposition-based
2.	the central nervous system	56	content	Noun-based
3.	**parts of the brain**	55	content	Noun-based
4.	**one of the most**	53	Referential (focus)	Noun-based
5.	**on the other hand**	40	Discourse organizer (contrastive marker)	Preposition-based
6.	**are more likely to**	39	Stance (possibility)	Verb-based
7.	of the nervous system	39	Content	Preposition-based
8.	in the form of	38	Referential (intangible framing)	Preposition-based

[1] . Shared LBs are bolded

9.	for example if you	36	Referential (explanatory)	Preposition-based
10.	**of the brain and**	33	content	Preposition-based
11.	areas of the brain	31	Content	Noun-based
12.	**as a result of**	31	Referential (intangible framing)	Preposition-based
13.	a wide range of	30	Referential (quantification)	Noun-based
14.	play a role in	30	Referential (explanation)	Verb-based
15.	can be used to	29	Stance (possibility)	Verb-based
16.	**in terms of the**	28	Referential (intangible framing)	Preposition-based
17.	in the same way	28	Referential (asserting equivalence)	Preposition-based
18.	as well as the	27	Referential (elaboration)	Fragment
19.	**the rest of the**	27	Referential (focus)	Noun-based
20.	**the end of the**	26	Referential (place reference)	Noun-based
21.	**at the end of**	25	Referential (place refernce)	Preposition-based
22.	at the time of	25	Referential (time marker)	Preposition-based
23.	in a way that	25	Referential (elaboration)	Preposition-based
24.	**is one of the**	25	Referential (focus)	Verb-based
25.	it is important to	25	Stance (epistemic)	Verb-based
26.	is the study of	23	Referential (specification)	Verb-based
27.	**is an example of**	22	Referential (explanation)	Verb-based
28.	the American Psychological Association	22	Content	Noun-based
29.	in a variety of	21	Referential (intangible framing)	Preposition-based
30.	is based on the	21	Referential (specification)	Verb-based
31.	studies have shown that	21	Referential (evidential)	Verb-based
32.	**of the brain the**	20	content	Fragment
33.	**on the basis of**	20	Referential (intangible framing)	Preposition-based
34.	research has shown that	20	Referential (evidential)	Verb-based
35.	the surface of the	20	Referential (specification)	Noun-based
36.	in the brain and	19	Content	Preposition-based

37.	in the presence of	19	Referential (intangible framing)	Preposition-based
38.	**of the brain are**	19	Content	Verb-based
39.	the back of the	19	Referential (place reference)	Noun-based
40.	the results of the	19	Referential (evidential)	Noun-based
41.	the sound of a	19	Referential (specification)	Noun-based
42.	when it comes to	19	Referential (focus)	Verb-based
43.	**in the absence of**	18	Referential (intangible framing)	Preposition-based
44.	more likely to be	18	Referential (imprecision)	Verb-based
45.	the effects of the	18	Discourse (causal relationship)	Noun-based
46.	**the middle of the**	18	Referential (place reference)	Noun-based
47.	in the left hemisphere	17	Content	Preposition-based
48.	the study of the	17	Referential (focus)	Noun-based
49.	**in the middle of**	16	Referential (place reference)	Preposition-based
50.	**of the brain is**	16	Content	Verb-based
51.	to a variety of	16	Referential (intangible framing)	Preposition-based
52.	to the development of	16	Referential (specification)	Preposition-based
53.	to the study of	16	Referential (specification)	Preposition-based
54.	at the beginning of	15	Discourse organizer (place reference)	Preposition-bsed
55.	in this case the	15	Referential (specification)	Preposition-based
56.	**that there is a**	15	Referential (specification)	Verb-based
57.	the fact that the	15	Stance (epistemic)	Noun-based
58.	the nervous system is	15	Content	Verb-based
59.	**the part of the**	15	Referential (focus)	Noun-based
60.	**the size of the**	15	Referential (tangible framing)	Noun-based
61.	in terms of their	14	Referential (intangible framing)	Preposition-based
62.	nervous system and the	14	Content	Noun-based
63.	**other parts of the**	14	Referential (specification)	Noun-based
64.	than the sum of	14	Content	fragment
65.	the top of the	14	Referential (focus)	Noun-based
66.	**to be able to**	14	Stance (ability)	Ver-based

67.	of the human brain	13	Content	Preposition-based
68.	participants were asked to	13	Content	Verb-based
69.	the left and right	13	Content	Noun-based
70.	the sum of its	13	Content	Noun-based
71.	were more likely to	13	Referential (imprecision)	Verb-based
72.	functional magnetic resonance imaging	12	Content	Noun-based
73.	in a number of	12	Referential (quantification)	Preposition-based
74.	in a series of	12	Referential (sequential processes)	Preposition-based
75.	is the result of	12	Referential (framing)	Verb-based
76.	sum of its parts	12	Content	Noun-based
77.	to the spinal cord	12	Content	Preposition-based
78.	a small number of	11	Referential (quantification)	Noun-based
79.	**is part of the**	11	Referential (specification)	Verb-based
80.	the form of a	11	Referential (intangible)	Noun-based
81.	the human brain is	11	Content	Verb-based
82.	various parts of the	11	Referential (quantification)	Noun-based
83.	areas of the cortex	10	Content	Noun-based
84.	**but it is not**	10	Referential (exception)	Verb-based
85.	for example in the	10	Referential (explanatory)	Preposition-based
86.	in one of the	10	Referential (focus)	Preposition-based
87.	in the human brain	10	Content	Preposition-based
88.	is controlled by the	10	Referential (specification)	Verb-based
89.	left and right hemispheres	10	Content	Noun-based
90.	play an important role	10	Referential (explanation)	Verb-based
91.	the right hemisphere is	10	Content	Verb-based
92.	was one of the	10	Referential (focus)	Verb-based
93.	a large number of	9	Referential (quantification)	Noun-based
94.	by the fact that	9	Stance (epistemic)	Preposition-based
95.	can be explained by	9	Stance (possibility)	Verb-based
96.	magnetic resonance imaging FMRI	9	Content	Noun-based
97.	**of the fact that**	9	Stance (epistemic)	Preposition-based
98.	plays a role in	9	Referential (explanation)	Verb-based

99.	sometimes referred to as	9	Referential (focus)	Verb-based
100.	**that the brain is**	9	Content	Verb-based
101.	and the effects of	8	Discourse (causal relationship)	Fragment
102.	as well as a	8	Discourse organizer (additional)	Preposition-based
103.	has a number of	8	Referential (quantification)	Verb-based
104.	**to look at the**	8	Referential (focus)	Verb-based
105.	how do we know	7	Stance (reader engagement)	Verb-based
106.	less likely to be	7	Referential (imprecision)	Verb-based
107.	may or may not	7	Stance (possibility/probability)	Fragment
108.	**one part of the**	7	Referential (specification)	Noun-based
109.	should be able to	7	Stance (ability)	Verb-based
110.	the limbic system the	7	Content	Fragment
111.	the rate at which	7	Referential (quantifying frequency)	Noun-based
112.	the two hemispheres of	7	Content	Noun-based
113.	to the central nervous	7	Content	Preposition-based
114.	two hemispheres of the	7	Content	Noun-based
115.	as part of a	6	Referential (focus)	Preposition-based

Appendix (C)

No	Lexical bundle	Freq	Function	Grammar structure
1.	I am going to	302	Stance (intention)	verb-based
2.	we are going to	226	Stance (intention)	verb-based
3.	part of the brain	216	Content	noun-based
4.	you are going to	170	Stance (prediction)	verb-based
5.	what is going on	154	Stance (listener engagement)	verb-based
6.	of the brain that	98	content	preposition-based
7.	there is a lot	97	Referential (quantification)	verb-based
8.	you do not have	92	Stance (listener engagement)	verb-based
**9.**	**parts of the brain**	**89**	**content**	**noun-based**
10.	we will talk about	88	Stance (intention)	verb-based
11.	it is kind of	86	Referential (specification)	verb-based
12.	is a lot of	86	Referential (quantification)	verb-based
13.	so here is the	86	Discourse (topic introduction)	verb-based
14.	if you do not	72	Stance (desire)	verb-based
15.	so here is a	71	Referential (topic introduction)	verb-based
16.	going to talk about	70	Stance (intention)	verb-based
17.	it is going to	67	Stance (prediction)	verb-based
**18.**	**is going to be**	**65**	**Stance (prediction)**	**verb-based**
19.	you do not know	65	Stance (epistemic)	verb-based
20.	and there is a	63	Referential (specification)	verb-based
21.	do not want to	62	Stance (lack of desire)	verb-based
22.	it turns out that	62	Stance (unexpected outcome)	verb-based
23.	do not have to	60	Stance (lack of obligation)	verb-based

24.	a little bit about	59	Referential (quantification)	noun-based
25.	and here is the	58	Discourse organizer (connecting)	verb-based
26.	they are going to	58	Stance (intention)	verb-based
27.	it is not a	57	Stance (evaluation & judgment)	verb-based
28.	a little bit of	56	Referential (quantification)	noun-based
**29.**	**of the brain and**	**56**	**Content**	**preposition-based**
30.	do not know what	55	Stance (uncertainty)	verb-based
**31.**	**on the other hand**	**55**	**Discourse organizer (contrastive marker)**	**preposition-based**
**32.**	**the part of the**	**55**	**Referential (focus)**	**noun-based**
33.	we do not know	55	Stance (uncertainty)	verb-based
34.	if you are a	54	Discourse organizer (conditional)	verb-based
**35.**	**is one of the**	**53**	**Referential (focus)**	**verb-based**
36.	are not going to	53	Stance (intention)	verb-based
37.	are going to talk	51	Stance (intention)	verb-based
38.	over and over again	50	Referential (repetition)	preposition-based
39.	a little bit more	49	Referential (quantification)	noun-based
**40.**	**at the same time**	**49**	**Referential (temporal coordination)**	**preposition-based**
41.	different parts of the	49	Referential (focus)	noun-based
**42.**	**in terms of the**	**47**	**Referential (intangible framing)**	**preposition-based**
43.	so it is not	47	Stance (judgment)	verb-based
44.	and it turns out	46	Stance (unexpected outcome)	verb-based
45.	if you have a	46	Discourse organizer (topic introduction)	verb-based
46.	if you want to	46	Stance (desire)	verb-based

**47.**	**that there is a**	**46**	**Referential (specification)**	**verb-based**
**48.**	**to be able to**	**46**	**Stance (ability)**	**verb-based**
49.	it is not just	45	Stance (emphasis)	verb-based
50.	a part of the	44	Referential (focus)	noun-based
51.	a lot of the	43	Referential (quantification)	noun-based
52.	are going to be	43	Stance (intention)	verb-based
53.	you look at the	43	Discourse organizer (topic introduction)	verb-based
54.	and it is not	42	Stance (judgment)	verb-based
55.	and one of the	42	Referential (focus)	noun-based
**56.**	**one of the most**	**42**	**Referential (focus)**	**noun-based**
57.	that is going to	42	Stance (intention)	verb-based
58.	it is a very	41	Stance (emphasis)	verb-based
59.	so I am going	41	Stance (intention)	verb-based
**60.**	**the end of the**	**40**	**Referential (place reference)**	**noun-based**
61.	are going to get	39	Stance (intention)	verb-based
62.	so here is what	39	Discourse organizer (topic introduction)	verb-based
**63.**	**the rest of the**	**39**	**Referential (focus)**	**noun-based**
64.	to do with the	39	Referential (indicating relationship)	verb-based
65.	when you look at	39	Discourse organizer (topic focus)	verb-based
66.	those of you who	38	Discourse organizer (specifying sub-group)	noun-based
67.	to figure out what	38	Discourse organizer (introducing a process of discovery)	verb-based
68.	when we talk about	38	Discourse organizer (topic introduction)	verb-based
69.	but there is a	37	Referential (specification)	verb-based
70.	it does not matter	37	Stance (emphasizing irrelevance)	verb-based
71.	you have got the	37	Referential (focus)	verb-based
72.	and this is a	36	Referential (identification)	verb-based
**73.**	**of the brain is**	**36**	**Content**	**verb-based**

74.	one of the things	36	Referential (focus)	noun-based
75.	are going to do	36	Stance (intention)	verb-based
76.	so that is the	36	Discourse organizer (highlighting important points)	verb-based
77.	you have got to	36	Stance (obligation)	verb-based
78.	and it is a	35	Referential (topic introduction)	verb-based
79.	and that is what	35	Referential (topic clarification)	verb-based
**80.**	**at the end of**	**35**	**Referential (place marker)**	**preposition-based**
81.	that is kind of	35	Stance (evaluation)	verb-based
82.	what you have got	35	Referential (identification)	verb-based
83.	you are looking at	35	Discourse organizer directing listeners attention)	verb-based
84.	but it is a	34	Referential (exception)	verb-based
85.	but it is not	34	Referential (exception)	verb-based
86.	if it is a	34	Referential (topic identification)	verb-based
87.	or something like that	34	Referential (imprecision)	verb-based
88.	in the United States	33	Referential (place reference)	preposition-based
89.	are going to see	33	Stance (prediction)	verb-based
90.	the idea is that	33	Referential (intangible framing)	verb-based
91.	what you do is	33	Discourse organizing (describing a procedure)	verb-based
92.	all of a sudden	32	Discourse organizing (unexpected even)	noun-based
93.	here is an example	32	Referential (focus)	verb-based
94.	if you think about	32	Discourse marker (directing attention to a topic)	verb-based
95.	going to be a	31	Stance (intention)	verb-based
96.	it is not the	31	Referential (exception)	verb-based

97.	am going to talk	31	Stance (intention)	verb-based
98.	we are talking about	31	Referential (topic focus)	verb-based
99.	you want to know	31	Stance (desire)	verb-based
100.	not going to be	30	Stance (intention)	verb-based
101.	right in front of	30	Discourse marker (driving the attention of the listener)	fragment
**102.**	**as a result of**	**29**	**Referential (intangible framing)**	**preposition-based**
103.	I do not want	29	Stance (desire)	verb-based
104.	I want you to	29	Stance (desire)	verb-based
105.	we do not have	29	Stance (lack of understanding)	verb-based
106.	you are not going	29	Stance (prediction)	verb-based
107.	do not have a	28	Stance (absence of specific outcome)	verb-based
108.	do not know how	28	Stance (epistemic)	verb-based
109.	I do not think	28	Stance (epistemic)	verb-based
110.	if you look at	28	Discourse organizer (guiding the reader)	verb-based
111.	so we are going	28	Stance (intention)	verb-based
112.	thank you very much	28	Politeness (special function)	verb-based
113.	what we are going	28	Discourse organizer (announcing future topics)	verb-based
114.	you do not get	28	Stance (absence of a specific outcome)	verb-based
115.	you do not want	28	Stance (lack of desire)	verb-based
116.	and you do not	27	Stance (epistemic)	verb-based
117.	I am not going	27	Stance (lack of intention)	verb-based
118.	is kind of a	27	Stance (hedging)	verb-based
119.	is not going to	27	Stance (intention)	verb-based
120.	trying to figure out	27	Discourse organizing (effort to understand something)	verb-based

121.	it is the same	26	Referential (asserting equivalence)	verb-based
122.	will talk about that	26	Stance (intention)	verb-based
123.	for those of you	25	Discourse organizing (specifying sub-group)	fragment
124.	having to do with	25	Referential (establishing relationship between concepts or processes)	verb-based
125.	it is just a	25	Stance (downplaying a concept or condition)	verb-based
126.	am not going to	25	Stance (intention)	verb-based
127.	that you do not	25	Referential (specification)	fragment
128.	what they do is	25	Discourse organizer (help introduce a topic)	verb-based
129.	to give you a	24	Stance (intention)	verb-based
130.	want to talk about	24	Stance (desire)	verb-based
131.	what I want to	24	Stance (desire)	verb-based
132.	a number of different	23	Referential (quantification)	noun-based
133.	in the real world	23	Referential (specification)	preposition-based
134.	of the things that	23	Referential (specification)	preposition-based
135.	that it is not	23	Referential (expressing negation or denial)	verb-based
136.	that is not the	23	Referential (expressing negation or denial)	verb-based
137.	the brain and the	23	Content	fragment
138.	this is a very	23	Referential (identification)	verb-based
139.	turns out to be	23	Stance (unexpected outcome)	verb-based
140.	you can see it	23	Discourse organizer (topic focus)	verb-based

141.	come up with a	22	Referential (generating ideas or solution)	verb-based
142.	for a long time	22	Referential (time reference)	preposition-based
143.	have a lot of	22	Referential (quantification)	verb-based
144.	I want to do	22	Stance (desire)	verb-based
145.	in the middle of	22	Referential (place reference)	preposition-based
146.	it is a good	22	Stance (epistemic)	verb-based
147.	parts of the body	22	content	noun-based
148.	they do not have	22	Referential (absence of certain qualities)	verb-based
149.	you have to have	22	Referential (signaling necessity or requirement)	verb-based
150.	as far as we	21	Stance (epistemic)	fragment
151.	at the level of	21	Referential (focus)	preposition-based
152.	but they do not	21	Referential (contrastive)	verb-based
153.	I will give you	21	Stance (intention)	verb-based
154.	is a little bit	21	Referential (quantification)	verb-based
155.	so this is the	21	Referential (topic introduction)	verb-based
**156.**	**the size of the**	**21**	**Referential (tangible framing)**	**noun-based**
157.	you do not see	21	Referential (absence of perception)	verb-based
158.	and it does not	20	Stance (contrastive)	verb-based
159.	that this is a	20	Referential (identification)	verb-based
160.	the brain that is	20	content	verb-based
161.	the middle of the	20	Referential (place reference)	noun-based
162.	we have talked about	20	Stance (engagement)	verb-based
163.	you think about it	20	stance (epistemic)	verb-based
164.	and so on and	19	Discourse (continuative marker)	fragment
165.	but you do not	19	Stance (contrastive)	verb-based
166.	of you who are	19	Referential (group identification)	verb-based

167.	is going on here	19	Stance (intention)	verb-based
168.	that is what we	19	Discourse (explanatory)	verb-based
169.	there is not a	19	Stance (expressing absence)	verb-based
170.	to look at the	19	Referential (focus)	verb-based
171.	we talked about the	19	Discourse (referring back to previous discussion)	verb-based
172.	what is the difference	19	Discourse (comparison)	verb-based
173.	you can see the	19	Stance (evidential marker)	verb-based
174.	a huge amount of	18	Referential (quantification)	noun-based
175.	and there is no	18	Stance (expressing absence	verb-based
176.	for the first time	18	Referential (time reference)	preposition-based
177.	over and over and	18	Referential (repetition)	fragment
178.	Not have to be	18	Stance (epistemic)	verb-based
179.	we are not going	18	Stance (absence of intention)	verb-based
180.	you want to do	18	Stance (desire)	verb-based
181.	and over and over	17	Referential (repetition)	fragment
182.	and then there is	17	Discourse (sequential additive marker)	verb-based
183.	and we do not	17	Stance (uncertainty)	verb-based
184.	and what happens is	17	Discourse (sequential)	verb-based
185.	and you can not	17	Stance (denial)	verb-based
186.	but it turns out	17	Stance (unexpected outcome)	verb-based
187.	I will talk about	17	Stance (intention)	verb-based
188.	if you have to	17	Discourse (conditional marker)	verb-based
189.	are more likely to	17	Stance (possibility)	verb-based
190.	is a very good	17	Stance (evaluative)	verb-based
191.	so you have to	17	Discourse (explaining a sequence of events)	verb-based

192.	that is a very	17	Stance (evaluative)	verb-based
193.	that is how you	17	Discourse (illustrative marker)	verb-based
194.	you do not need	17	Stance (absence of necessity)	verb-based
195.	you are trying to	17	Stance (intention)	verb-based
196.	a picture of a	16	Referential (exemplification)	noun-based
197.	are a lot of	16	Referential (quantification)	verb-based
198.	in the absence of	16	Referential (intangible framing)	preposition-based
199.	is that there is	16	Referential (identification)	verb-based
200.	it is in the	16	Referential (location marker)	verb-based
201.	let us look at	16	Discourse (attention-driving marker)	verb-based
**202.**	**is an example of**	**16**	**Referential (explanatory)**	**verb-based**
203.	that you can not	16	Stance (undercutting assumption)	verb-based
204.	there are a lot	16	Referential (quantification)	verb-based
205.	there is a very	16	Stance (evaluative)	verb-based
206.	you have to do	16	Discourse (procedural marker)	verb-based
207.	and that is not	15	Stance (evaluative)	verb-based
208.	he is going to	15	Stance (intention)	verb-based
209.	in front of the	15	Referential (place marker)	preposition-based
**210.**	**of the brain the**	**15**	**Content**	**preposition-based**
211.	that we do not	15	Stance (negation)	verb-based
212.	there has to be	15	Stance (obligation)	verb-based
213.	there is going to	15	Stance (intention)	verb-based
214.	what I am going	15	Stance (intention)	verb-based
215.	what you are doing	15	Discourse (attention-driving)	verb-based
216.	and you will see	14	Discourse (expectation)	verb-based
217.	at the university of	14	Referential (place marker)	preposition-based

218.	but that is not	14	Discourse (contrastive)	verb-based
219.	Does not seem to	14	Stance (degree of uncertainty)	verb-based
220.	in terms of how	14	Referential (intangible framing)	preposition-based
221.	not going to get	14	Stance (prediction)	verb-based
222.	one or the other	14	Discourse (making contrasting options)	noun-based
223.	so you do not	14	Stance (providing instructions/guidanc e)	verb-based
224.	that if you are	14	Stance (conditional)	verb-based
225.	that is involved in	14	Discourse (focus)	verb-based
226.	the front of the	14	Referential (place)	noun-based
227.	the question is how	14	Referential (topic exploration)	verb-based
228.	what you want to	14	Stance (desire)	verb-based
229.	you can have a	14	Stance (possibility)	verb-based
230.	you are in a	14	Stance (engagement)	verb-based
231.	and it is the	13	Referential (focus)	verb-based
232.	and you want to	13	Stance (desire)	verb-based
233.	does not have to	13	Stance (lack of obligation)	verb-based
234.	figure out how to	13	Stance (problem-solving tech)	verb-based
235.	have to do is	13	Discourse (give instructions)	verb-based
236.	if you are in	13	discourse (conditional)	verb-based
237.	in a couple of	13	Referential (quantification)	preposition-based
238.	in different parts of	13	Referential (focus)	preposition-based
239.	it does not make	13	Stance (denial)	verb-based
240.	it turns out there	13	Stance (unexpected outcome)	verb-based
**241.**	**of the brain are**	**13**	**Content**	**verb-based**
242.	of the world and	13	Referential (place)	preposition-based
243.	so we do not	13	Stance (denial)	verb-based

244.	so what happens is	13	Discourse (sequential)	verb-based
245.	the level of the	13	Referential (focus)	noun-based
246.	there is a big	13	Referential (specification)	verb-based
247.	they are kind of	13	Referential (specification)	verb-based
248.	turned out to be	13	Stance (unexpected outcome)	verb-based
249.	and then you have	12	Discourse (sequential)	verb-based
250.	and there is some	12	Referential (focus)	verb-based
251.	are not going to	12	Stance (intention)	verb-based
252.	because there is a	12	Discourse (causal relation marker)	verb-based
253.	because there is no	12	Discourse (causal relation marker	verb-based
254.	go back to the	12	Discourse (attention-driving)	verb-based
255.	going to focus on	12	Stance (intention)	verb-based
256.	I think that is	12	Stance (epistemic)	verb-based
257.	if there is a	12	Discourse (conditional)	verb-based
258.	if you have any	12	Discourse (conditional)	verb-based
259.	is an example the	12	Referential (explanatory)	verb-based
260.	it is a really	12	Referential (focus)	verb-based
261.	it is not as	12	Stance (comparative evaluation)	verb-based
**262.**	**on the basis of**	**12**	**Referential (intangible framing)**	**preposition-based**
263.	so if you are	12	Discourse (conditional)	verb-based
264.	the fact that you	12	Stance (epistemic)	noun-based
265.	the one that is	12	Referential (focus)	verb-based
266.	there is a little	12	Referential (quantification)	verb-based
267.	There has been some	12	Referential (quantification)	verb-based
268.	to get out of	12	Discourse (indicating escape/exit)	verb-based

269.	today we are going	12	Stance (intention)	verb-based
270.	what happens is the	12	Discourse (sudden outcome	verb-based
271.	what they are doing	12	Stance (intention)	verb-based
272.	you are thinking about	12	Stance (epistemic)	verb-based
273.	but if you are	11	Discourse (conditional)	verb-based
274.	but you can see	11	Stance (contrastive)	verb-based
275.	but you are not	11	Stance (contrastive)	verb-based
276.	Can not do that	11	Stance (lack of ability)	verb-based
277.	do not have any	11	Stance (absence of qualities)	verb-based
278.	do not need to	11	stance (absence of a requirement)	verb-based
279.	has a lot of	11	Referential (quantification)	verb-based
280.	has to be a	11	Stance (requirement or necessity)	verb-based
281.	have to worry about	11	Stance (expressing concerns)	verb-based
282.	here you have got	11	discourse (attention-driving)	verb-based
283.	how is it that	11	Stance (listener engagement)	verb-based
284.	in the case of	11	Referential (intangible framing)	preposition-based
285.	in the first place	11	discourse (drawing attention to initial conditions)	preposition-based
286.	in the history of	11	Referential (framing)	preposition-based
287.	in the size of	11	Referential (intangible framing)	preposition-based
288.	is that it is	11	Referential (focus)	verb-based
289.	it does not work	11	Stance (signalling ineffectiveness or failure)	verb-based
290.	it is one of the	11	Referential (focus)	verb-based
291.	one part of the	11	Referential (specification)	verb-based
292.	is a part of	11	Referential (focus)	verb-based
293.	is not just that	11	Stance (emphasizing complexity)	verb-based

294.	so if you have	11	Discourse (conditional)	verb-based
295.	so that is what	11	Discourse (explanation)	verb-based
296.	that is a little	11	Referential (quantification)	verb-based
297.	that is part of	11	Referential (focus)	verb-based
298.	that the brain is	11	content	verb-based
299.	the shape of the	11	Referential (framing)	noun-based
300.	they tend to be	11	Stance (intention)	verb-based
301.	to be the case	11	Referential (focus)	verb-based
302.	to think about it	11	Stance (epistemic)	verb-based
303.	you can think of	11	Stance (epistemic)	verb-based
304.	an example of this	10	Referential (explanatory)	noun-based
305.	and what they did	10	Discourse (topic elaboration)	verb-based
306.	but it is also	10	Discourse marker (introducing further information)	verb-based
307.	but the idea is	10	Discourse (establishing contrastive relations)	verb-based
308.	going to try to	10	Stance (intention)	verb-based
309.	had a lot of	10	Referential (quantification)	verb-based
310.	I would like to	10	Stance (intention)	verb-based
311.	I think there is	10	Stance (epistemic)	verb-based
312.	if you had a	10	Discourse (conditional)	verb-based
313.	is a picture of	10	Referential (framing)	verb-based
314.	it is part of	10	Referential (specification)	verb-based
315.	know there is a	10	Referential (specification)	verb-based
316.	of a lot of	10	Referential (quantification)	preposition-based
317.	of course it is	10	Stance (epistemic)	verb-based
**318.**	**other parts of the**	**10**	**Referential (specification)**	**noun-based**
**319.**	**is part of the**	**10**	**Referential (specification)**	**verb-based**

320.	is the difference between	10	Referential (contrastive)	verb-based
321.	the beginning of the	10	Referential (place reference)	noun-based
322.	the fact that we	10	Stance (epistemic)	noun-based
323.	there is no way	10	Referential (intangible framing)	verb-based
324.	this a little bit	10	Reference (quantification)	fragment
325.	what happens in the	10	Stance (outcomes)	verb-based
326.	what you need to	10	Stance (necessity)	verb-based
327.	when there is a	10	Referential (time marker)	verb-based
328.	won the Nobel prize	10	Content	verb-based
329.	you think about the	10	Stance (epistemic)	verb-based
330.	a little bit later	9	Referential (time reference)	noun-based
331.	a lot of you	9	Referential (quantification)	noun-based
332.	and there was a	9	Referential (specification)	verb-based
333.	behavior in terms of	9	Referential (intangible framing)	fragment
334.	but they are not	9	Referential (contrastive)	verb-based
335.	does not really matter	9	Stance (downplaying a claim)	verb-based
336.	I am not sure	9	Stance (epistemic)	verb-based
337.	if I do not	9	Discourse (conditional)	verb-based
338.	if that is the	9	Discourse (conditional)	verb-based
339.	if you are going	9	Discourse (conditional)	verb-based
340.	if you are not	9	Discourse (conditional)	verb-based
341.	is a kind of	9	Referential (specification)	verb-based
342.	is that you can	9	Stance (ability)	verb-based
343.	it was kind of	9	Referential (specification)	verb-based
344.	is a lot more	9	Referential (quantification)	verb-based

345.	not have to do	9	Stance (negation)	verb-based
346.	not know what is	9	Stance (epistemic)	verb-based
347.	that is what I	9	Stance (epistemic)	verb-based
348.	that we can not	9	Stance (lack of ability)	verb-based
349.	that we are going	9	Stance (intention)	verb-based
350.	the same time and	9	Referential (time reference)	fragment
351.	then there is a	9	Referential (specification)	verb-based
352.	they are more likely	9	Referential (imprecision)	verb-based
353.	what happens to the	9	Discourse (attention-drawing)	verb-based
354.	what happens when you	9	Discourse (sequential)	verb-based
355.	you are kind of	9	Stance (engagement)	verb-based
356.	a couple of years	8	Referential (time reference)	noun-based
357.	as far as I	8	Stance (epistemic)	fragment
358.	because you can not	8	Stance (judgement)	verb-based
359.	by looking at the	8	Discourse organizer (topic focus)	preposition-based
360.	Did not want to	8	Stance (desire)	verb-based
361.	do not do it	8	stance (negation of action)	verb-based
362.	do not see it	8	Stance (negation of perception)	verb-based
363.	for a lot of	8	Referential (quantification)	preposition-based
364.	get a lot of	8	Referential (quantification)	verb-based
365.	going to be able	8	Stance (prediction)	verb-based
366.	if any of you	8	Discourse (conditional)	fragment
367.	it is called the	8	Referential (specification)	verb-based
368.	it turned out that	8	Stance (unexpected outcome)	verb-based
369.	am going to do	8	Stance (intention)	verb-based
370.	not going to do	8	Stance (intention)	verb-based
371.	now I want to	8	Stance (desire)	verb-based

**372.**	**of the fact that**	**8**	**Stance (epistemic)**	**preposition-based**
373.	so we have a	8	Stance (ability)	verb-based
374.	so what we are	8	Discourse (transition)	verb-based
375.	talk about that in	8	Stance (intention)	verb-based
376.	that is what it	8	Discourse (conclusion)	verb-based
377.	that there is something	8	Referential (quantification)	verb-based
378.	that we talked about	8	Stance (listener engagement)	verb-based
379.	the brain is the	8	Content	verb-based
380.	that brain that is	8	Content	verb-based
381.	the first part of	8	Referential (specification)	noun-based
382.	the first thing you	8	Discourse (sequential)	noun-based
383.	the same as the	8	Discourse (comparison)	noun-based
384.	the way you do	8	Referential (intangible framing)	verb-based
385.	this is actually a	8	Referential (identification)	verb-based
386.	this is the only	8	Referential (specification)	verb-based
387.	to be afraid of	8	Stance (epistemic)	verb-based
388.	to think about the	8	Stance (epistemic)	verb-based
389.	what the brain does	8	Content	verb-based
390.	why do not you	8	Stance (listener engagement)	verb-based
391.	you can see here	8	Stance (listener engagement)	verb-based
392.	you can not get	8	Stance (listener engagement)	verb-based
393.	you do not really	8	Stance (listener engegment)	verb-based
394.	you know you are	8	Stance (listener engagement)	verb-based
